# A new ophthalmosaurid ichthyosaur from the Upper Jurassic (Early Tithonian) Kimmeridge Clay of Dorset, UK, with implications for Late Jurassic ichthyosaur diversity

**DOI:** 10.1371/journal.pone.0241700

**Published:** 2020-12-09

**Authors:** Megan L. Jacobs, David M. Martill

**Affiliations:** 1 School of the Environment, Geography and Geosciences, University of Portsmouth, Portsmouth, United Kingdom; 2 Department of Geosciences, Baylor University, Waco, TX, United States of America; Chinese Academy of Sciences, CHINA

## Abstract

A new ophthalmosaurid ichthyosaur, *Thalassodraco etchesi* gen. et sp. nov., from the Upper Jurassic Kimmeridge Clay Formation of Dorset, UK is described. The specimen, a partial, articulated skull and anterior thorax in the Etches Collection of Kimmeridge, Dorset, is exceptionally well preserved on a slab of laminated coccolith limestone and has been expertly prepared. It comprises a near complete skull in articulation with associated anterior vertebral column and dorsal ribs, complete pectoral girdle, fully exposed left forelimb, and some elements of the right forelimb. Other elements present, including an ischiopubis are preserved on separate slabs. Presumed rapid burial of the anterior portion of the specimen in the coccolith substrate has preserved a number of ossified ligaments lying across the vertebral column and associated ribs as well as stomach contents and decayed internal organs. Aspects of the dentition, skull roof bones and the forelimb configuration distinguishes the new specimen from previously described Late Jurassic ichthyosaurs. Autopmorphies for *T*. *etchesi* include a large rounded protuberance on the supratemporal bone; a thin L-shaped lachrymal, with a steeply curved posterior border; ~ 70 teeth on the upper tooth row, and deep anterior dorsal ribs. A well resolved phylogenetic analysis shows *T*. *etchesi* as a member of a basal clade within Ophthalmosauridae comprising *Nannopterygius*, *Gengasaurus*, *Paraophthalmosaurus* and *Thalassodraco*. The new specimen adds to the diversity of the Ichthyopterygia of the Kimmeridge Clay Formation and emphasises the important contribution of amateur collectors in palaeontology.

## Introduction

Ichthyosaurs were a successful group of large marine reptiles for most of the Mesozoic. They first appeared in the Early Triassic (Olenekian), ~248 million years ago [1], and became extinct in the early Late Cretaceous (Cenomanian), approximately 90 million years ago [2–5] By the Jurassic, Ichthyosaurs had evolved into highly adapted marine predators, with a streamlined body for moving through the water [6, 7], large eyes for improved vision at depth [8] and an elongated skull with jaws full of conical teeth, suited for catching fish and squid [9–11]. Well preserved ichthyosaurs are known from four Jurassic deposits in the British Isles, the Early Jurassic Blue Lias and Whitby Mudstone formations, the middle Jurassic Peterborough Member of the Oxford Clay Formation and the Upper Jurassic Kimmeridge Clay Formation of Dorset [12]. These formations have long been known to yield marine reptiles, especially ichthyosaurs, and have become of immense historical significance [13–17].

The Upper Jurassic Kimmeridge Clay Formation exposed along the Dorset coastline at Kimmeridge Bay is famous for yielding exceptionally preserved vertebrates, including ichthyosaurs [18, 19]. A new museum in the village of Kimmeridge houses the now famous, but largely unstudied Etches collection that includes several new and important ichthyosaur specimens [20].

The Etches collection opened to the general public in 2016, purposely built to house the lifetime collection of Dr Steve Etches MBE, who collected almost exclusively from the Kimmeridge Clay Formation for over 30 years. The collection contains many ichthyosaurs, including several articulated specimens and numerous isolated skull bones, vertebrae, girdle elements and fore and hind limbs. The majority of these specimens remain unstudied and several appear, at first glance, new to science.

**Fig 1:**
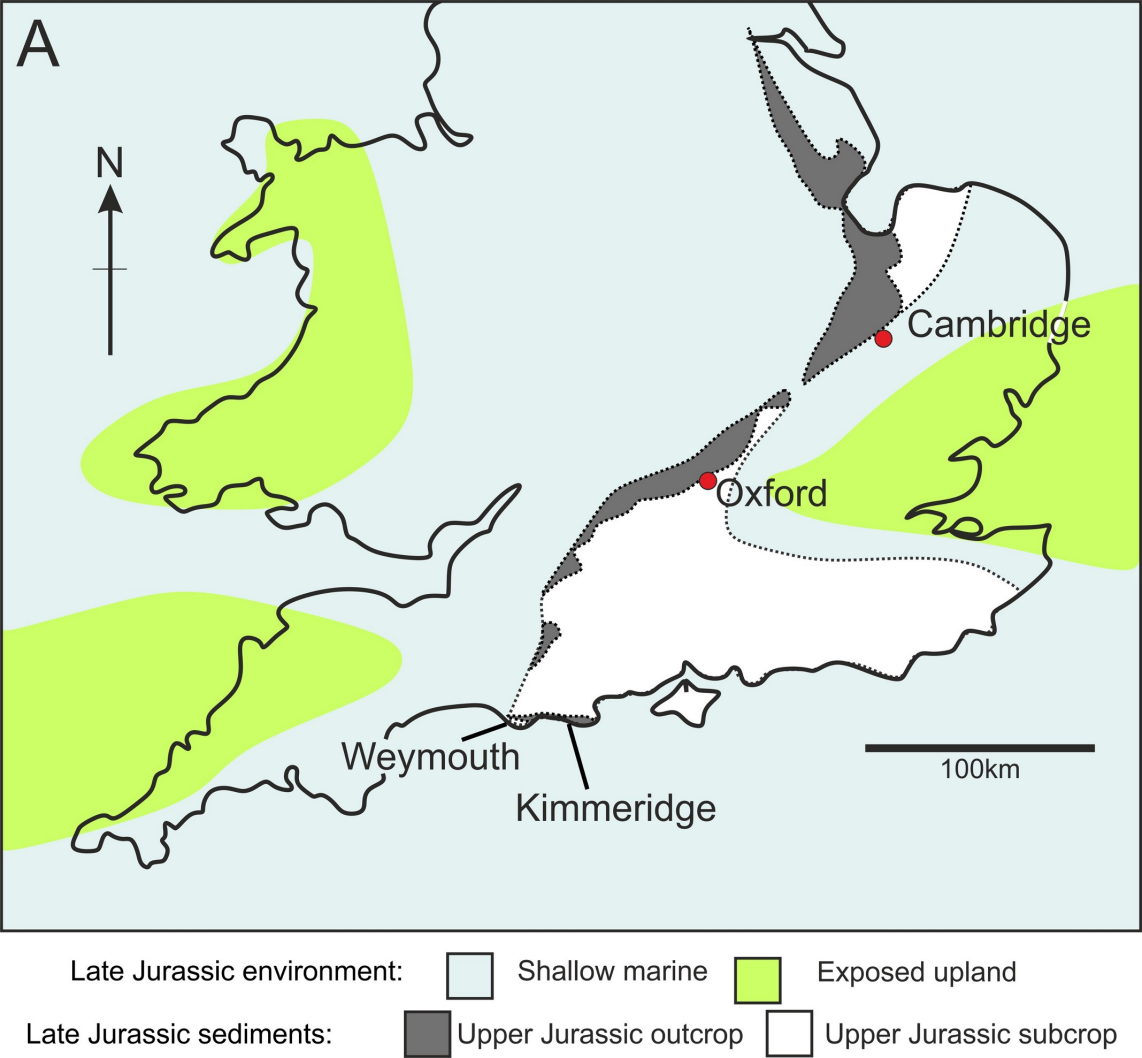
A, Outcrop and subcrop map of the onshore Upper Jurassic Kimmeridge Clay Formation, showing the locality of Kimmeridge on the English South Coast, and sea coverage over the United Kingdom during the Late Jurassic. Outcrop and sea coverage data after Martill *et al*. [21], and Foffa *et al*. [22].

**Fig 2:**
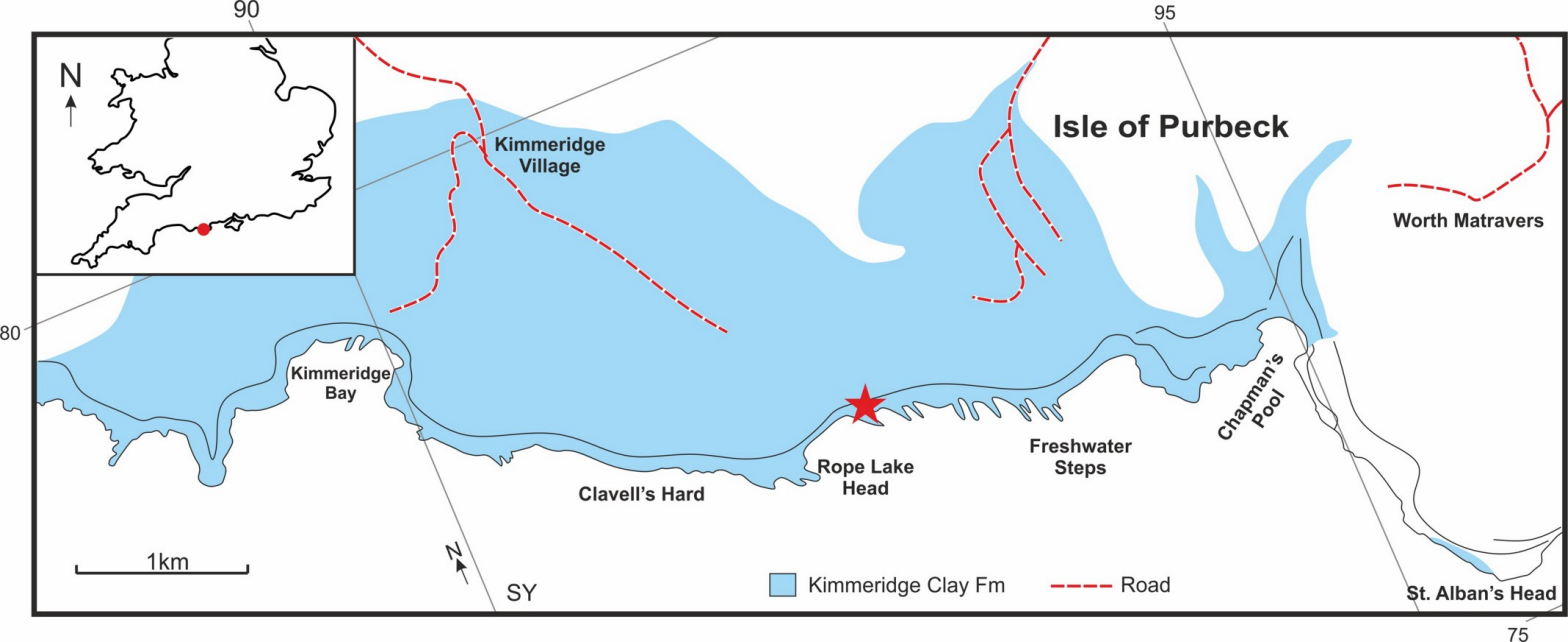
Map showing the Kimmeridge Clay Formation outcrop in Dorset, southern England. The White Stone Band is exposed between Rope Lake Head and Freshwater Steps. Star indicates the locality of MJML K1885. Base map data from Lees *et al*. [23].

Here we describe a well preserved specimen (MJML K-1885) collected in 2009 by Dr Etches from the White Stone Band outcropping in Rope Lake Bay, Dorset (Figs 1 and 2). Based on a unique combination of features MJML K-1885 is referred to a new taxon, *Thalassodraco etchesi*.

### Geological setting

The Late Jurassic Kimmeridge Clay Formation crops out in a narrow strip from the Dorset coast to Yorkshire, with outcrops also present on the East coast of Scotland and the Isle of Skye (Fig 1A) [24–26]. The type section is the cliffs and foreshore sequence exposed at Kimmeridge Bay, Dorset [24]. In Dorset, the formation crops out at three localities along this stretch of coastline; from St Aldhelm’s Head to Kimmeridge Bay and Gad Cliff (OS Grid. Ref: SY 89664–94767; from Ringstead Bay to Osmington Mills (OS Grid. Ref: SY 764815–735817) and around the northern margin of Portland Bill near Weymouth and eastern Fleet (OS Grid. Ref: SY 670765) (Fig 2). Over the past 200 years, the formation has become famous for yielding a wide diversity of fossil vertebrates, reported from over 60 localities across England [12], including several articulated specimens of bony and cartilaginous fishes, pliosaurs, ichthyosaurs, crocodiles, partial remains of pterosaurs and dinosaurs. It also yields a wide diversity of shelly fauna, especially cephalopods and a restricted assemblage of trace fossils [12, 20, 21, 27–31].

The new ichthyosaur specimen described here, MJML K-1885, was collected from fallen blocks of the ‘White Stone Band’, at National Grid Reference of SY932775 on the 14^th^ February 2009. This locality is situated between Rope Lake Head and Freshwater Steps and lies within the Jurassic Coast World Heritage Site (Fig 2).

The Kimmeridge Clay Formation in Dorset comprises a rhythmic sequence of soft mudstones, calcareous mudstones and kerogen-rich, black laminated shales with numerous thin limestone and dolostone horizons [32]. The onshore Kimmeridge Clay Formation was deposited in a shallow, epeiric sea [33], during the Kimmeridgian and early Tithonian stages of the Late Jurassic and was possibly subject to two major open ocean influences (Fig 1) [34]. During this time, there was a global sea level high stand [35], and levels of atmospheric carbon dioxide and temperatures were elevated [36–38] with widespread mudrock deposition across northwest Europe [39–41]. The thick sequence of bituminous shales and clays was deposited in calm bottom waters, with periods of anoxia in a stratified water column [29, 38, 41, 42]. The sediments of the Kimmeridge Clay are derived mostly from terrestrial environments, indicating substantial erosion from a nearby landmass [43]. However, plant macrofossils, excluding fossil wood, are rare, indicating that the deposition was some distance from the paleoshoreline [29].

**Fig 3:**
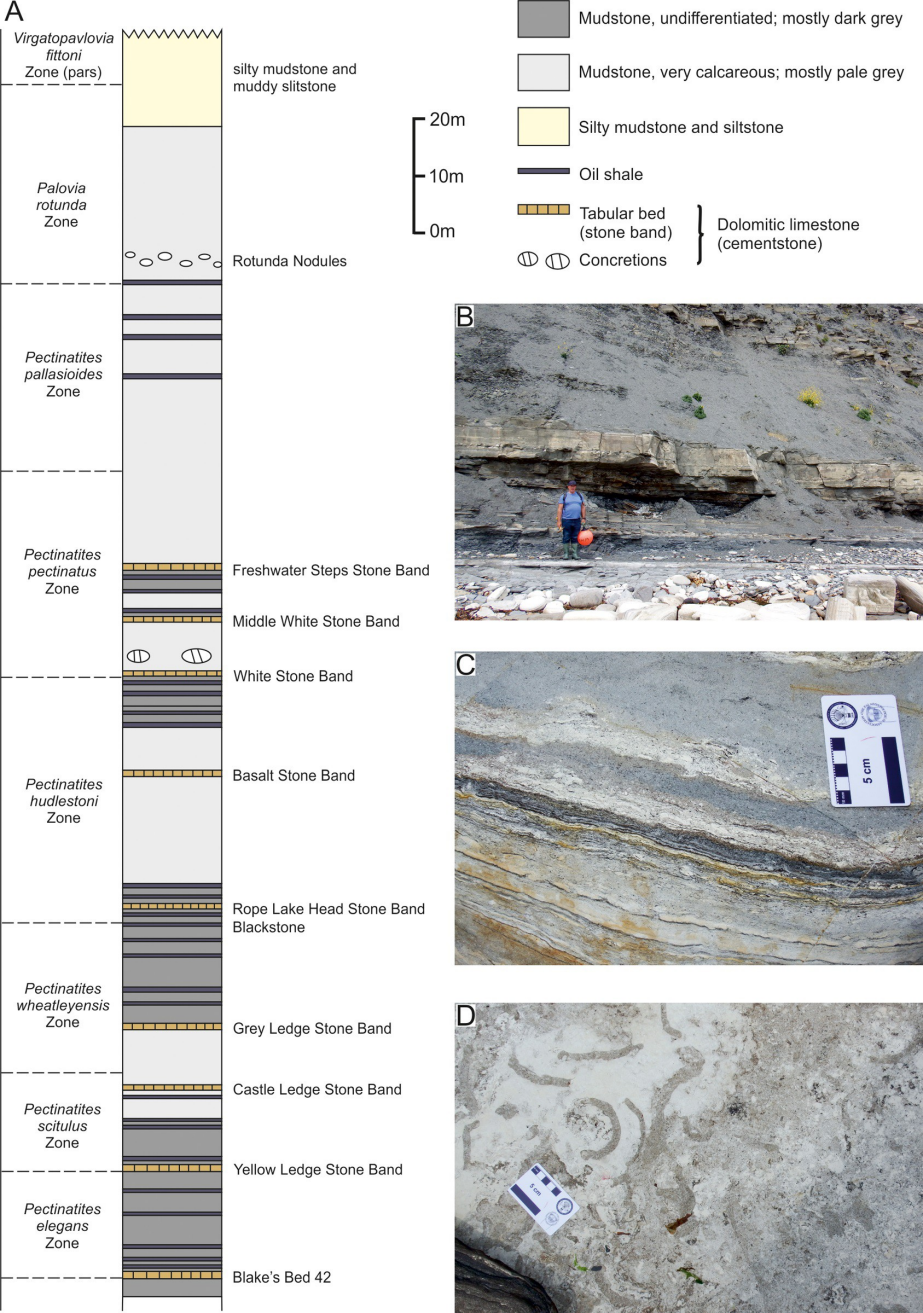
A, Generalised sedimentary log of the exposure from Kimmeridge Bay to Chapmans Pool, with ammonite zonation. Stratigraphy and ammonite zonation after Cox & Gallois [26]. B, exposure of the White Stone Band at beach level. C, laminae on a fallen block with an organic rich layer. D, bioturbation on a bedding surface on a fallen block. Scale bar for B, C and D represents 50 mm.

Towards the top of the formation at Rope Lake Head are 5 thin beds of pale coloured, laminated coccolith limestone (Fig 3), the thickest of which is known as the White Stone Band (bed number K46 of Gallois [44]). This conspicuous horizon is about 0.9 m thick and composed of over 100 laminae with a thin (~50 mm) oil shale towards its base. Other coccolith limestones in the sequence are generally less than 0.1 m thick [33]. These beds occur in the *Pectinatites pectinatus* ammonite zone, *eastlecottensis* subzone, of the lowermost Tithonian part of the sequence [45]. The White Stone Band only rarely yields tetrapods, with large and articulated vertebrates almost unknown (Etches, S, in pers. comm. 2019).

Detailed descriptions of the lithostratigraphy of the Kimmeridge Clay can be found in Cox & Gallois [26], Gallois & Etches [45], Morgans-Bell *et al*. [46] and Cope [47]. Detailed studies of the White Stone Band can be found in Gallois & Medd [33], Lees *et al*. [23] and Pearson *et al*. [48].

## Materials and methods

**Fig 4:**
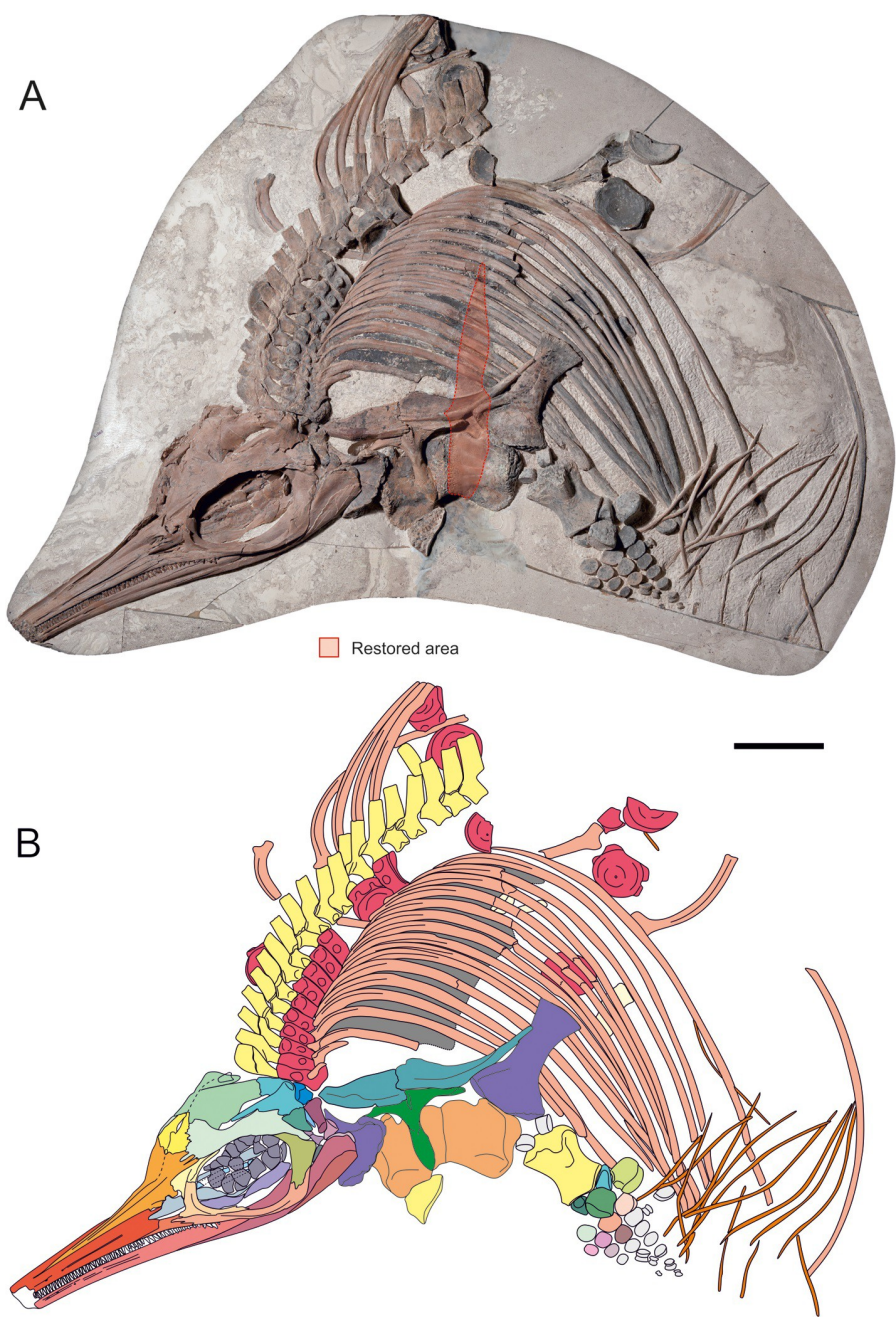
Anterior portion on the main block of *Thalassodraco etchesi*, MJML K 1885. A, photograph showing area restored during preparation. B, interpretive drawing of anterior portion of the skeleton. Scale bar represents 300 mm. Photograph used by permission of the Etches Collection under the CC BY 40 license, original copyright 2016.

Photographs were taken by MJ using a Fuji finepix Hs20 at the Etches Collection, Kimmeridge. Photographs in Figs 4, 7, 12 and 19 were provided by the Etches Collection under the CC BY 40 license, original copyright year 2016. All figures were made by MJ using Corel Draw x8.

### Nomenclatural acts

The electronic edition of this article conforms to the requirements of the amended International Code of Zoological Nomenclature, and hence the new names contained herein are available under that Code from the electronic edition of this article. This published work and the nomenclatural acts it contains have been registered in ZooBank, the online registration system for the ICZN. The ZooBank LSIDs (Life Science Identifiers) can be resolved and the associated information viewed through any standard web browser by appending the LSID to the prefix "http://zoobank.org/". The LSID for this publication is: urn:lsid:zoobank.org:pub:C1D99FF1-D4FC-4526-8E28-1B8FA64E21C0. The electronic edition of this work was published in a journal with an ISSN and has been archived and is available from the following digital repositories: PubMed Central, LOCKSS.

### Phylogenetic analysis

For the phylogenetic analysis, we used the matrix of Zverkov & Jacobs [49]. The data matrix consisted of 34 taxa and 112 characters. However only 68 of the characters could be coded for MJML K-1885, owing to the absence of the posterior portion of the specimen, excluding the ischiopubis, and loss of data caused by crushing thus obscuring the basicranium.

The dataset was compiled using MESQUITE v.3.61 [50] and the matrix was exported as a *.xlsx file (see S1 File). The analysis was performed in TNT v.1.5 [51], applying a traditional search with 10,000 replicates, tree bisection and reconnection with 100 trees saved per replication. Decay indices (Bremer support, optimal = 3) were also performed in TNT v. 1.5.

### Institutional abbreviations

CCMGE, Chernyshev’s Central Museum of Geological Exploration, Saint Petersburg, Russia; GLAHM, The Hunterian Museum, University of Glasgow, Glasgow, UK; IRSNB, Royal Belgian Institute of Natural Sciences, Brussels, Belgium; MJML, Museum of Jurassic Marine Life, Kimmeridge, Dorset, UK; NHMUK, Natural History Museum, London, UK; SGM, V.I. Vernadsky State Geological Museum of the Russian Academy of Sciences, Moscow, Russia; PMO, Nature History Museum, University of Oslo, Oslo, Norway; YKM, Ulyanovsk Regional Museum of Local Lore, Ulyanovsk, Russia.

## Results

### Systematic palaeontology

ICHTHYOSAURIA de Blainville, 1835 [52]
NEOICHTHYOSAURIA Sander, 2000 [53]
THUNNOSAURIA Motani, 1999 [54]
Family OPHTHALMOSAURIDAE Baur, 1887 [55]
Genus THALASSODRACO gen. nov.

**LSID**: urn:lsid:zoobank.org:act:E8B34A34-B932-4ECA-9DD5-E494482F20F2

**Derivation of generic name**: From *Thalasso*–(Gr) meaning sea, and—*draco* (Lat.) meaning dragon.

#### Type species

*Thalassodraco etchesi* gen. et sp. nov. (see below).

#### Diagnosis

As for the type and only species (see below).

Thalassodraco etchesi gen. et sp. nov.

Figs 4, 5, 7–17.

**Fig 5:**
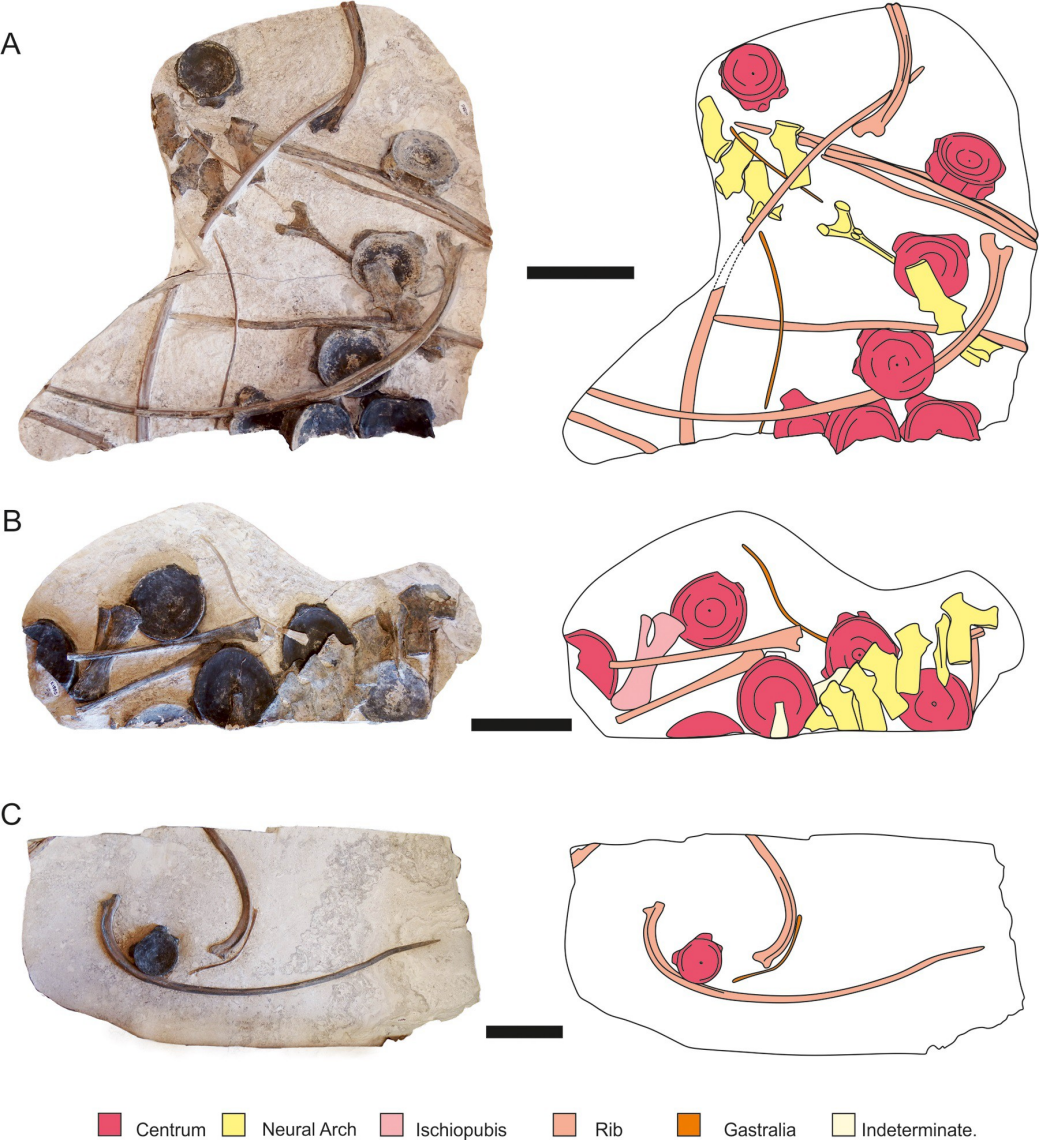
Isolated slabs of *Thalassodraco etchesi*. A, MJML K1885, B, MJML K1885, C, MJML K1896. Scale represents 100 mm.

**LSID**: urn:lsid:zoobank.org:act:35626449-D5AC-4970-B76E-27A827126D23

**Derivation of specific name:** named in honour of Dr Steve Etches MBE, who found and expertly prepared the specimen.

**Fig 6:**
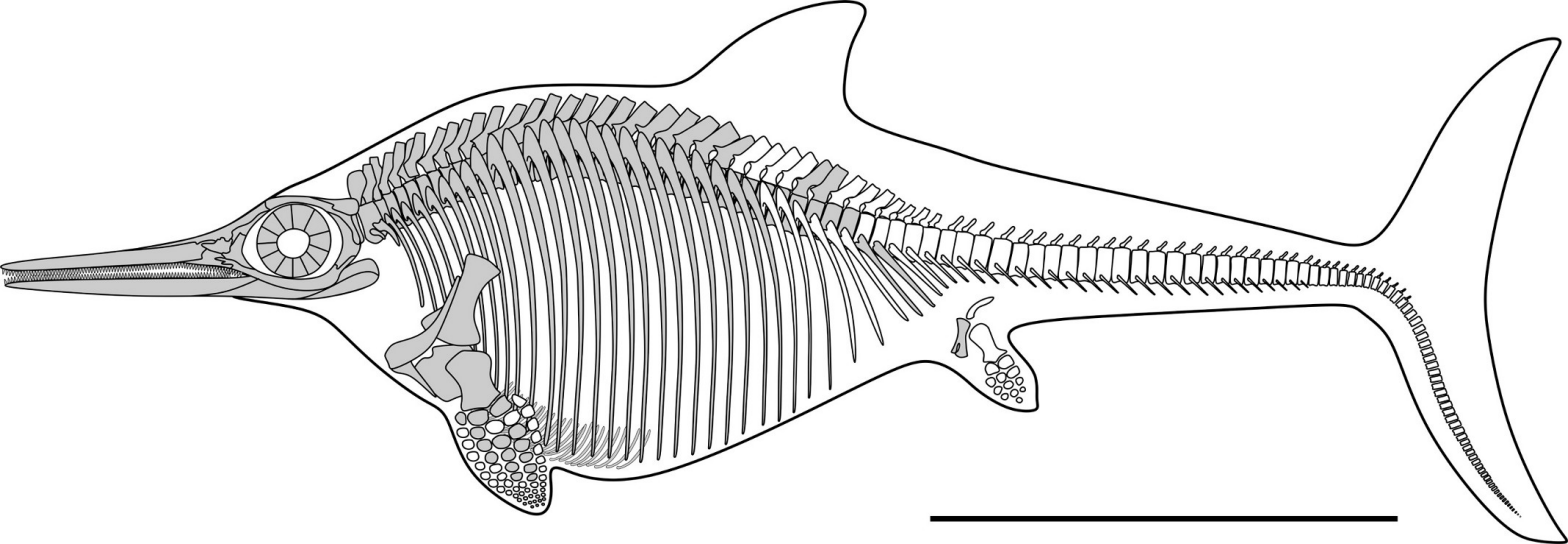
Skeletal reconstruction of *Thalassodraco etchesi* MJML K 1885. Grey areas indicating bones present. Scale represents 1 m.

**Fig 7:**
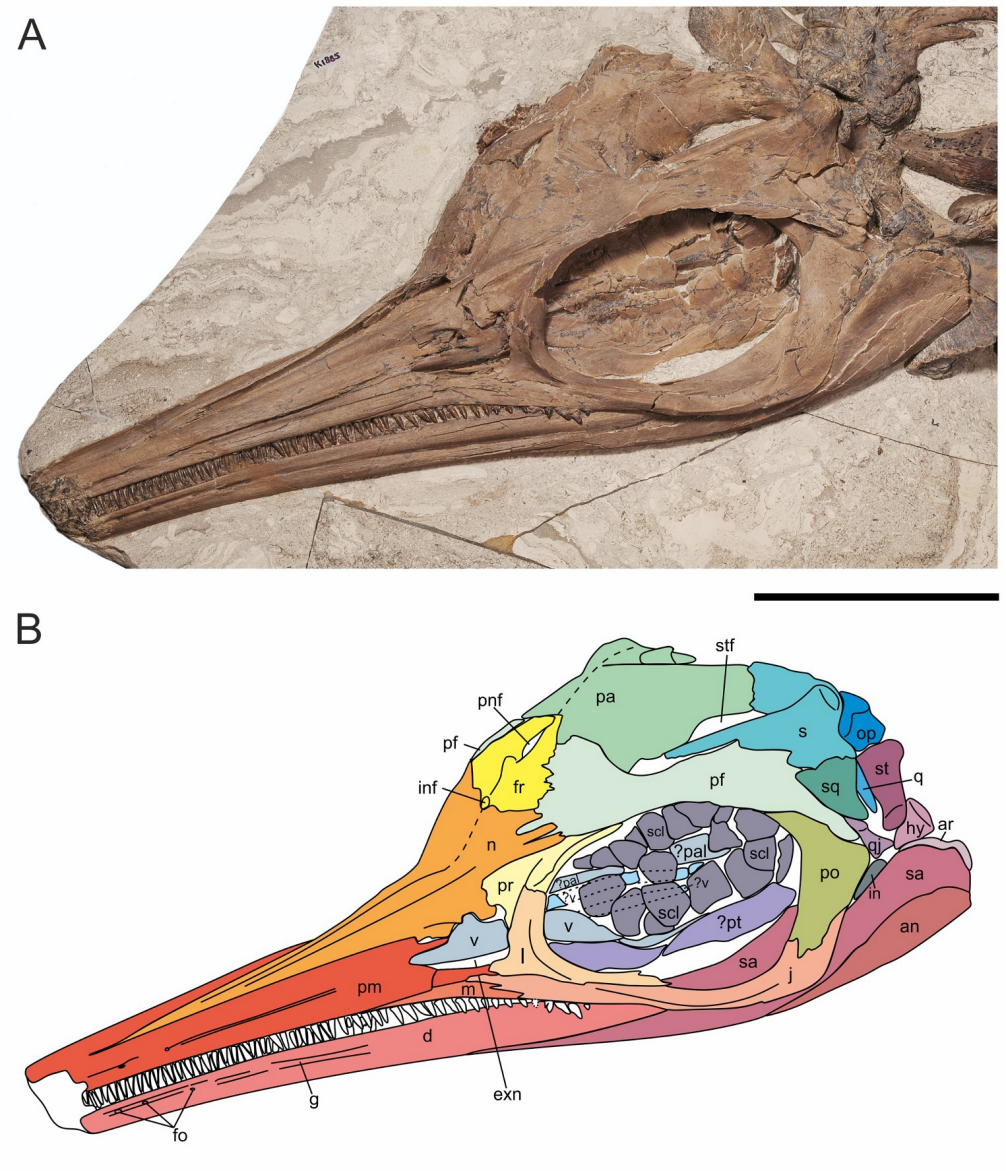
Skull of *Thalassodraco etchesi*, MJML K 1885. A, photograph of the holotype skull. B, interpretation of skull. Abbreviations: an, angular; ar, articular; d, dentary; exn, external naris; Fo, foramen; fr, frontals; G, groove;hy, hyoid; in, indeterminate; inf, internasal foramen; j, jugal; l, lachrymal; m, maxilla; n, nasal; op, opisthotic; pa, parietal; pal, palatine; pf, postfrontal; pnf, pineal foramen; pm, premaxillae; po, postorbital; pr, prefrontal; pt, pterygoid; q, quadrate; qj, quadratojugal; s, supratemporal; sa, surangular; scl, sclerotic ring; sq, squamosal; st, stapes; stf, supratemporal fenestra; v, vomer. Scale bar 100 mm. Photograph used by permission of the Etches Collection under the CC BY 40 license, original copyright 2016.

**Holotype:** MJML K1885, a partial articulated skeleton comprising the skull, pectoral girdle, left forelimb, anterior trunk and three isolated blocks with an ischiopubis and MJML K1896 a slab with an isolated rib and centrum (Figs 4–7).

**Referred specimen:** MJML K1174

**Type locality:** Rope Lake Bay, Kimmeridge, Dorset, UK. Grid reference SY932775.

**Type horizon and age:** White Stone Band, (Bed number K46 of Gallois [45]), Kimmeridge Clay Formation, *Pectinatites pectinatus* ammonite zone, Upper Jurassic, Early Tithonian.

*Diagnosis*. *Thalassodraco etchesi* is a medium sized ophthalmosaurid ichthyosaur (up to 2.25 m in maximum estimated length) characterised by the following autapomorphies (marked with a *) and unique character combinations: supratemporal bone with a enormously developed posterolateral tubera located posterodorsally and directed posterolaterally*; wide and robust postorbital with a straight anteroventral margin, with a sharp curve dorsally; jugal with a straight suborbital bar, a posterior dorsal upturn and an elongated dorsal process and dorsal ramus of jugal articulating exclusively with the postorbital in lateral view; a gracile L-shaped lachrymal, with a steeply curved posterior border* and a short anteroventral process extending to the external naris; estimated 70 teeth on the upper tooth row*; extremely short lateral exposure of the maxilla; deep anterior dorsal ribs, approximately 13 times as long as height of vertebral centrum; straight, rod-like distal portion of the clavicle; T- shaped interclavicle, with a spoon-shaped posterior ramus with a medial ridge on the anterior portion of posterior ramus (but not as spatulate as seen in *Grendelius* and *Undorosaurus*)*; scapular blade expands distally, giving overall hour-glass outline and proximally expanded with prominent acromion process*; concave posterior margins of the coracoid, a shallow anterior notch, and is expanded posteroventrally beyond the posterior border of the glenoid; anteromedial process of the coracoid moderately developed and appears to be rounded; medially placed, weakly developed dorsal trochanter of the humerus; reduced ventral process of the humerus, with no substantial protuberance in proximal view; rounded proximal and distal phalanges; a low number of phalanges with rapid distal constriction in size; ischiopubis fused only proximally, with an obturator foramen and the distal portions making contact, but unfused.

### Osteological description: Axial skeleton

#### Skull

The skull is exposed to reveal its left side, with most bones of the right side concealed or only partially exposed. Thus, most of the descriptions below are based on elements of the left side of the skull (Fig 7).

The left side of the skull is exposed and well preserved. Sutures on the dorsal portion are difficult to discern through crushing. The distal ends of the premaxilla and dentary are broken, so the full length of the skull cannot be measured, but it is estimated that approximately 100 mm are missing, suggesting a total skull length of ~520 mm. Estimated snout ratio (skull/ snout length) for MJML K1885 is 1.7. The orbit is slightly deformed due to crushing and measures 131 mm long and 85 mm high. It has an estimated orbital ratio (diameter of orbit/ length of lower jaw, Motani [54]) of 0.26 and a prenarial ratio (prenarial length/length of lower jaw) of 0.29. See S1 Table for measurements.

#### Premaxilla

The anterior extremities of the premaxillae have been eroded away, however the remaining portions are well preserved (Fig 7). The left premaxilla is well exposed, but due to compaction the right premaxilla has realigned exposing its medial surface and premaxilla-nasal suture. The premaxilla at the external naris is slightly crushed (Fig 7). The supranarial process is elongate and well developed, contributing to ~80% of the dorsal border of the external naris, although the distal portion is damaged but still present within the naris. The subnarial process extends further posteriorly, participating in the ventral border of the external naris and terminates in contact with the ventral margin of the lachrymal and jugal. Approximately 28 teeth are visible in the left premaxilla (Fig 7). Three anteroposteriorly elongated foramina with longest diameters between 3 and 9 millimetres are located dorsal to the tooth row along the anterior lateral margin of the premaxilla. The posterior-most foramina develop into a deep longitudinal groove extending posteriorly (fossa premaxillaris). The posteriormost margin of the premaxilla contacts the anterodorsal margin of the jugal. Crushing prevents determining the presence of a premaxilla-lachrymal contact. There is a clear contact with the anterior margin of the maxilla, with the maxilla located underneath the premaxilla, however the contact is unclear ventral to the external naris.

#### Maxilla

**Fig 8:**
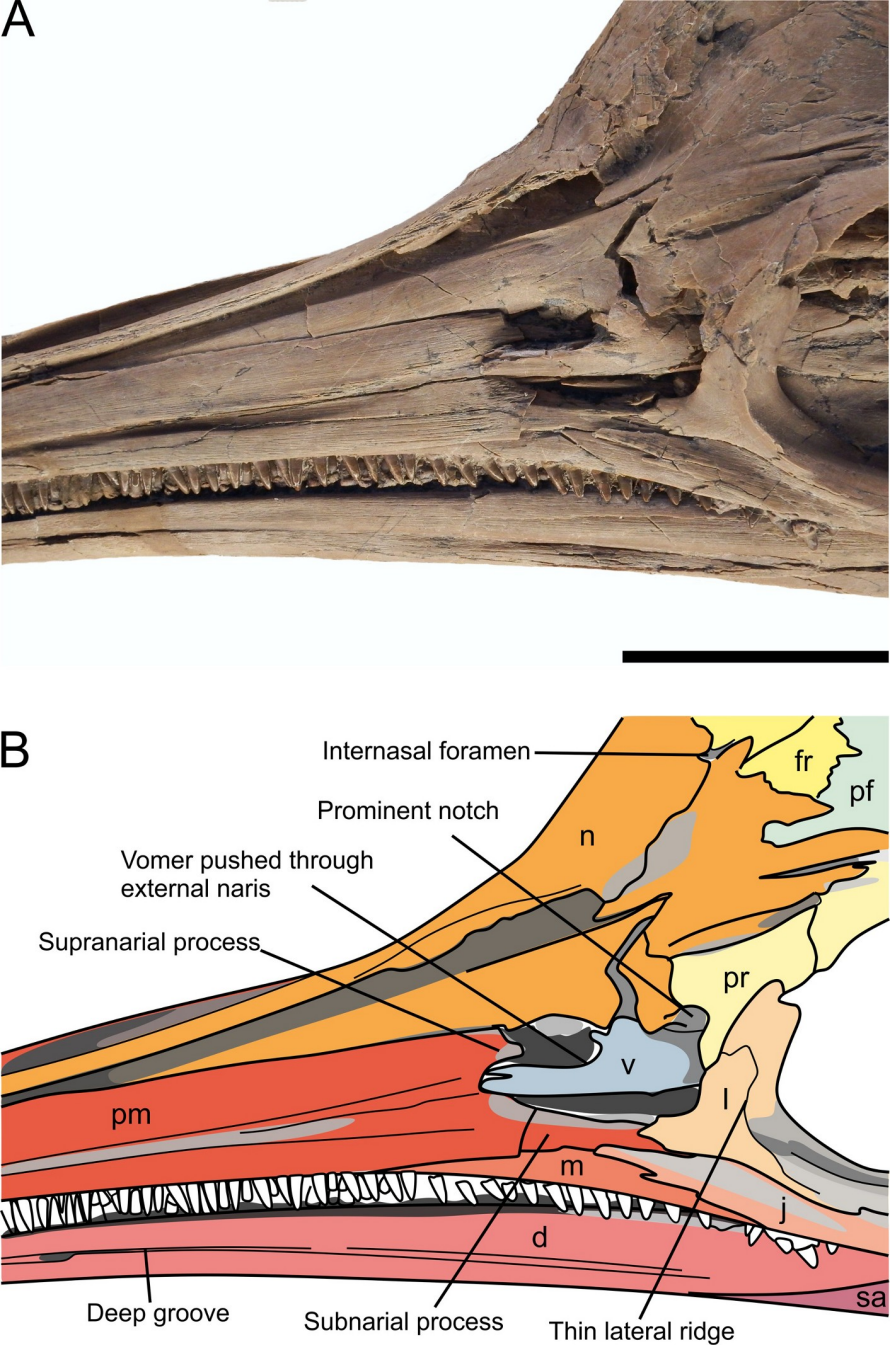
The anterior portion of the skull of *Thalassodraco etchesi* MJML K 1885. A, photograph of the anterior portion of the skull. B, annotated interpretation. See caption for Fig 7 for abbreviations. Scale bar 50 mm.

The left maxilla is poorly exposed and is mostly obscured by the premaxilla and jugal. It is excluded from the external naris by the premaxilla and jugal. There are 20 teeth visible in the maxilla (Fig 8). The maxilla contacts the lachrymal posterodorsally and contact with the jugal is extensive posteriorly. The exposed part of the maxilla elongates and tapers anteriorly with the premaxilla contact.

#### Lachrymal

**Fig 9:**
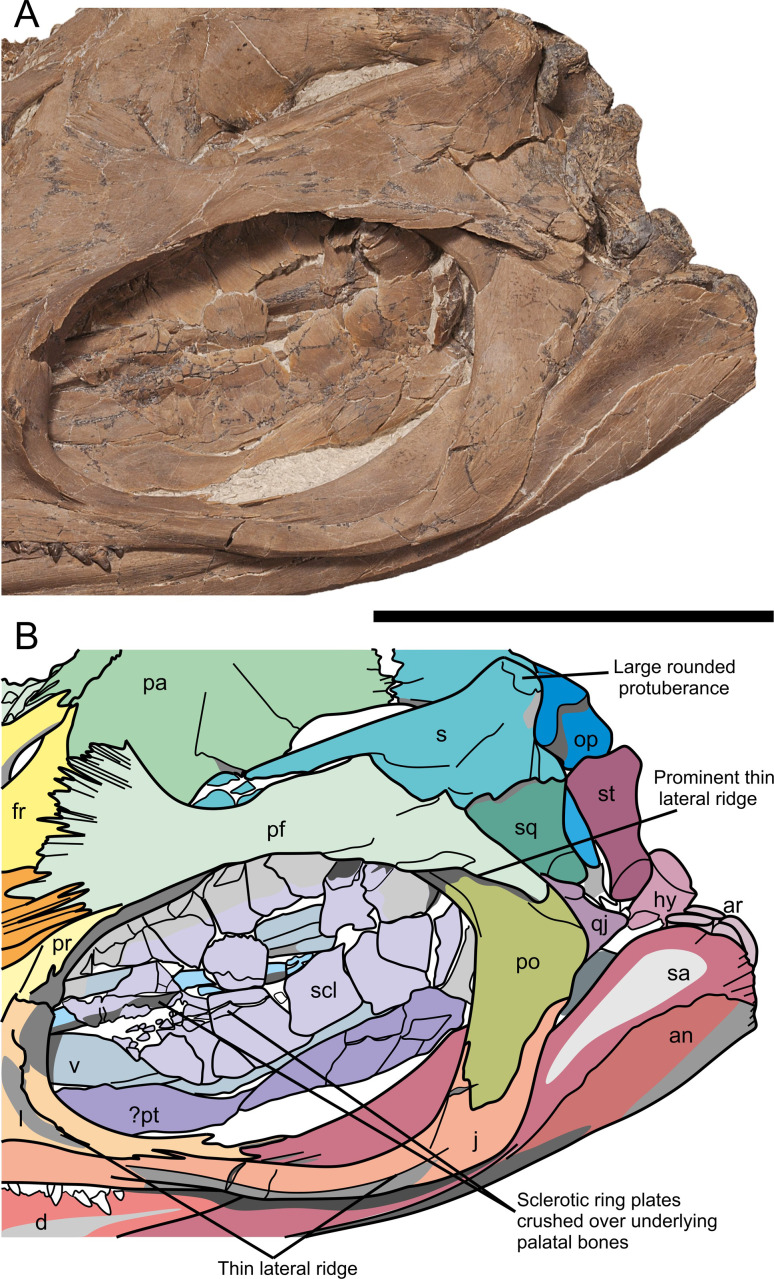
Posteroventral portion of the skull of *Thalassodraco etchesi* MJML K 1885. A, photograph of the posteroventral portion of the skull. B, annotated interpretation. See caption for Fig 7 for abbreviations. Scale bar 50 mm.

The lachrymal is well preserved (Figs 7 and 9). It contributes approximately 75% of the anterior margin, and nearly half of ventral margin of the orbit. The lachrymal contacts the posterior border of the external naris, with an anterior process contributing to part of the ventral border. Externally, the entire ventral margin contacts with the jugal. The dorsal border of the lachrymal ends abruptly with an sinusoidal suture with the prefrontal, distinctly lacking irregular interdigitation (Fig 8). The posterior margin, which forms the anteroventral border of the orbit is curved through an arc of approximately 115°. Its contribution to the orbital rim forms a prominent thin lateral ridge that slopes obliquely in the centre of the element and extends from its anteroventral border dorsally, similar to the condition seen in other ophthalmosaurids (Fig 8).

#### Jugal

The jugal is an elongate ‘J’-shaped element that contacts the lachrymal anterodorsally, the premaxilla and maxilla anteriorly and the postorbital posteriorly. The jugal becomes fan-like and thin anteriorly as it overlaps the maxilla. The suborbital bar of the jugal is narrow and robust, and is relatively straight along its length, bearing only a slight curve on the ventral border (Fig 9). The posteriormost portion is strongly curved dorsally through an arc of 107° relative to the suborbital bar. The jugal widens at the posterior margin and forms the ventral margin of the postorbital with an irregular suture. A lateral ridge extends along the mid-section of the jugal anteroposteriorly until the point of its curvature.

#### Nasals

**Fig 10:**
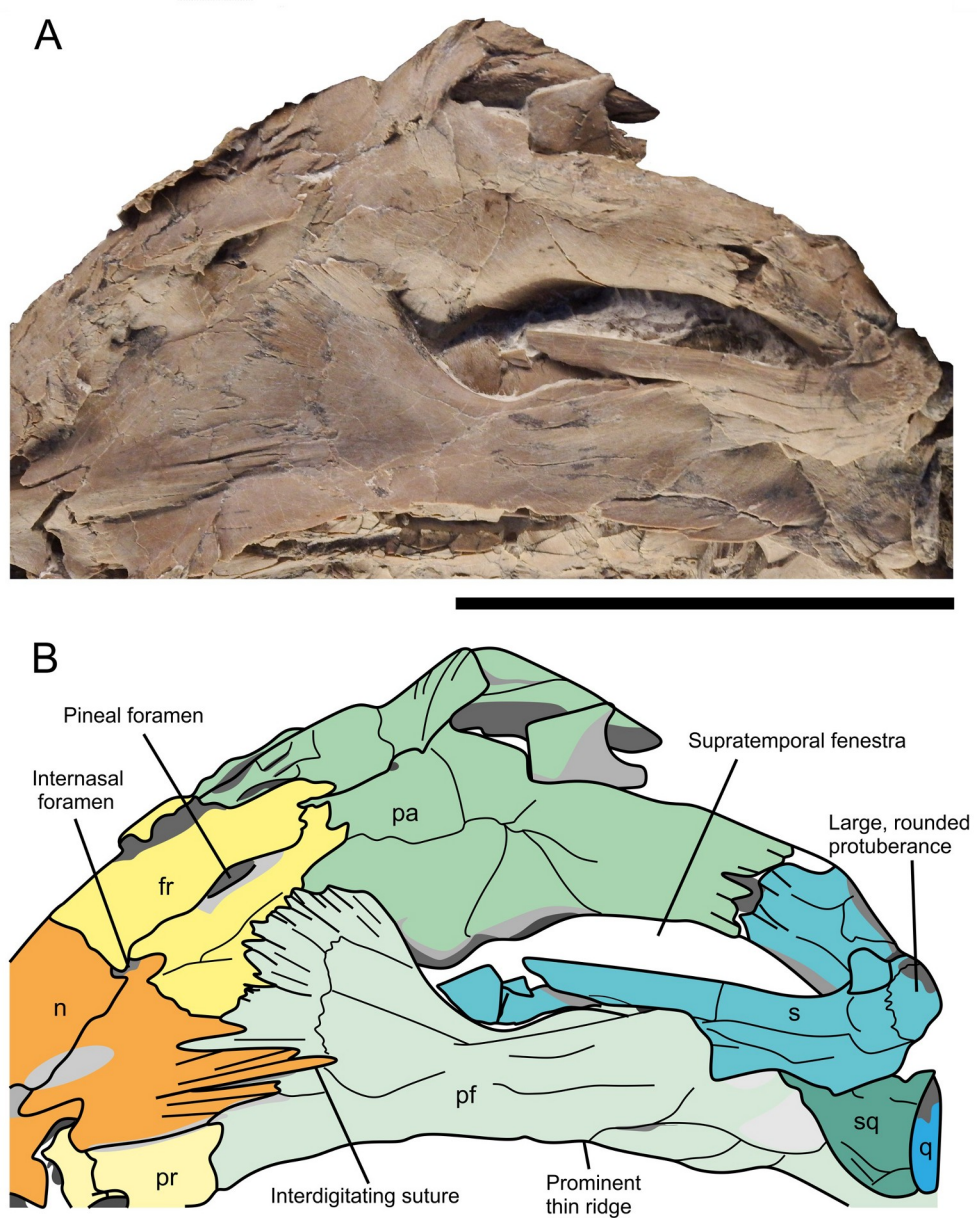
Dorsal portion of the skull of *Thalassodraco etchesi* MJML K 1885. A, photograph of the dorsal portion of the skull. B, annotated interpretation. See caption for Fig 7 for abbreviations. Scale bar 50 mm.

The left nasal is well exposed and well preserved, with only slight crushing in its posterior portion near the internasal foramen (Figs 7 and 9). The right nasal is present but mostly obscured by the right premaxilla. The nasal is curved laterally towards the midline, which would have formed a rostrum with a smooth curve. The nasal forms the majority of the border of a small internasal foramen. There is a pronounced dorsal depression (excavatio internasalis) surrounded by ridges laterally. The internasal foramen is located posteromedially of the excavatio internasalis. Posteriorly, the nasal contacts the anterior borders of the postfrontals and frontal along a zone of well-defined interdigitating processes (Figs 7 and 10). The nasal is overlain by the prefrontal, with a suture ill-defined due to crushing.

#### Prefrontals

The prefrontal is a triangular element, strongly dorsoventrally compressed posteriorly, overlain by the postfrontals and nasals posteriorly (Fig 7). The contact with the lachrymal is sinusoidal on the orbit rim, and is obscured by a wide anterior process, that overlays the anterodorsal portion of the lachrymal and the nasal. There is a ventrally extending process, contributing to the dorsoposterior border of the external naris with a prominent notch (Fig 8). The prefrontal contributes to approximately one third of the dorsal margin of the orbit. It bears an obliquely sloping, prominent thin lateral ridge, extending anteroventrally to posterodorsally, and forms a continuation of the ridge seen on the lachrymal. This ridge may have supported the dermal covering protecting the eye [56].

#### Frontals

The frontals are approximately triangular in dorsal outline, bordering the nasals anteriorly and have the anterior 75% of their lateral border contacting the postfrontals with an interdigitating suture (Fig 7). Approximately 75% of the posterior border margin contacts the parietals, but the midline suture cannot be distinguished anteriorly due to crushing. Two long posterior processes surround the pineal foramen (Fig 10) and overlie the parietals anteromedially with an interdigitating suture. There is no contribution of the frontal to the supratemporal fenestra. A small internasal foramen (~ 5 mm diameter) is present at the frontal–nasal contact.

#### Postfrontals

The postfrontals are large, prominent elements contributing to approximately 66% of the dorsal margin of the orbit (Fig 7). The anterior part articulates with the nasals, frontals and parietals along a zone of well-defined interdigitating processes, contributing to almost the whole of the frontals’ lateral margin (Fig 10). The postfrontals extend posteriorly, contacting the supratemporal, squamosal and postorbital with an ill-defined margin due to crushing. The anterior ventral margin is markedly curved and contributes to almost the entirety of the anterior border of the supratemporal fenestra but is obscured from the lateral border by the supratemporal. The lateral portion of the postfrontals becomes sheet-like posteriorly, with a prominent thin ridge extending along the orbit margin, which is a continuation of a ridge on the lachrymal and prefrontal. The ridge extends anteroposteriorly, over the postorbital.

#### Parietals

The left parietal is well preserved and exposed in dorsolateral view, while the right parietal is preserved, but mostly obscured by lateral crushing of the skull. The left parietal is a broad bone with a strongly curved lateral margin, where it forms the medial margin of the supratemporal fenestra (Fig 10). The two posterior processes of the frontals overlie the parietals anteromedially. There is a small articulation with the postfrontals on the dorsomedial margin, distal to the frontals, with large interdigitating sutures. The supratemporal process of the parietals articulates with the medial ramus of the supratemporal with a straight suture with small interdigitations along the margins. The length of medial contact is comparatively elongate, and the posteromedial notch is absent (cf. *Arthropterygius*). The supratemporal process is moderately long, but not as robust as in some platypterygiines. There is no evidence of ornamentation or a sagittal crest along the dorsal surface of the skull.

#### Postorbitals

The postorbital contribute to the entirety of the posterior margin of the orbit, extending onto the dorsal margin, beneath the lateral ridge of the postfrontal (Fig 7). There is a small ridge on the anterodorsal surface extending posteroventrally for 76 mm. Dorsally, the postorbital is rod-like and slightly compressed dorsoventrally, with a wide anteroposterior expansion in the mid-section. It then narrows ventrally, where it contacts the jugal with a large ventral process, forming an ‘S’ shape sutural contact (Fig 9). The postorbital contacts the quadratojugal at the point of maximum anteroposterior expansion. The postorbital is excluded from contact with the supratemporal by the postfrontal. The articulation with the postfrontal is broad, with the suture obscured by the prominent lateral ridge (Fig 9).

#### Supratemporals

**Fig 11:**
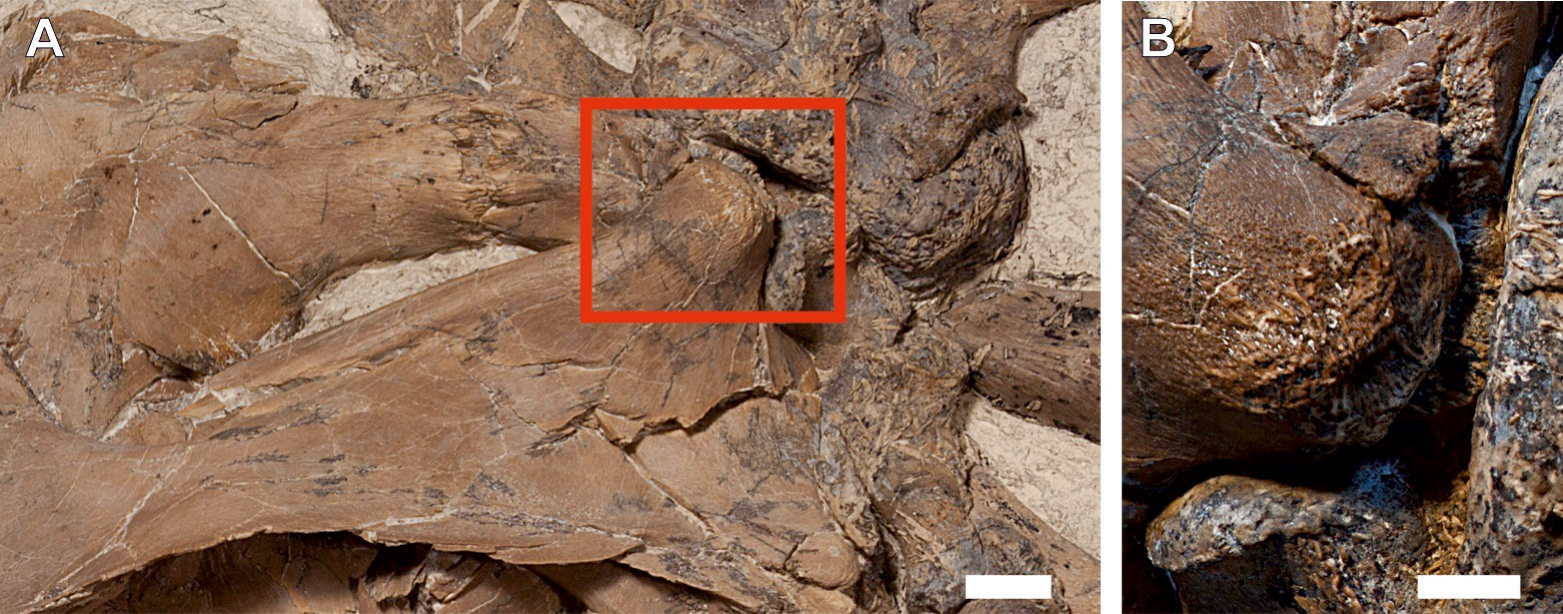
Supratemporal bone of *Thalassodraco etchesi*, MJML K 1885. A, posterodorsal portion of the skull, with red box indicating the protuberance of the supratemporal. B, rugose texture on the supratemporal protuberance. Scale bar A, 10 mm; B, 5 mm.

The supratemporal bones form the posterior and lateral margins of the supratemporal fenestra (Fig 7). The medial ramus of the supratemporal is short and robust, articulating with the supratemporal process of the parietal. Externally, the anterior ramus is mediolaterally compressed distally and rod-like anteriorly. The anterior most portion of the supratemporal is broken, so the nature of its anterior margin and its extent is unknown. The anterior ramus has a broad posteroventral expansion, which contacts the squamosal, however, due to crushing, this contact is poorly defined. On the posterodorsal portion of the supratemporal is the ‘posterolateral tubera’ [18]; a prominent and well developed, rounded process, directed posterolaterally (Fig 10). The process is covered in a heavy rugose texture, suggesting an attachment point for an extensive cartilage or for the *depressor mandibulae muscle* (Fig 11). It is unclear how prominent these would have been in life, but with extensive cartilage present it is possible that two eminences were visible.

#### Squamosal

The squamosal is a small triangular element articulating with the postfrontal in a straight margin, and no anterior expansion (Figs 7 and 10). The squamosal contacts the posteroventral margin of the supratemporal, but the exact nature of the contact is unclear. There is also contact with the quadratojugal, but the extent of this contact cannot be determined.

#### Quadratojugal

The quadratojugal is small with highly reduced lateral exposure. Its posterior margin is gently concave, but all other margins are obscured by overlapping elements of the lateral skull (Fig 9).

#### Other cranial elements

Opisthotic and stapes identities have been tentatively assigned to two exposed posterior elements, based on the cranial morphology of other ophthalmosaurids [16, 18] (Fig 8). Mostly these elements are partially obscured due to the lateral crushing of the skull and obscured by adjacent bones.

#### Opisthotic

The opisthotic is party obscured by the supratemporal, but that visible shows it to have a short and robust paraoccipital process.

#### Stapes

This bone is moderately well exposed and compares well with the same element in *Ophthalmosaurus icenicus* from the Oxford Clay Formation [18], displaying a moderate shaft, with sub triangular facet for the quadrate.

### Lower jaw

#### Dentary

The left dentary is well preserved and visible in left lateral view. The right dentary is not visible, being obscured by the left dentary. The dentary is missing approximately 100 mm of the anterior end due to erosion. The dentary gently tapers from posterior to anterior. The ventral margin bears a very slight concavity in its middle portion, and the dorsal margin also has a corresponding convexity. The dentary bears 35 visible teeth (Fig 7), with an estimated 53 in total for the preserved portion of the dentary (approximately 18 teeth are either missing or obscured by teeth of the premaxilla/maxilla). In addition, it is estimated that the missing portion of the dentary could have carried 20 teeth, giving a minimum tooth count of approximately 73. Towards the anterior end of the dentary is a single row of four large (diameter = 3 mm) foramina, which develop into a deep groove that extends posteriorly and continues for the majority of the length of the dentary parallel to the tooth row. The dentary contacts the surangular posteriorly with a straight and well-defined border (Fig 9).

#### Surangular

The left surangular is well preserved and elongate, with the anterior most margin extending as far as the anterior border of the external naris. In lateral view, posteriorly it is wide, and tapers anteriorly to a sharp point beneath the dentary. The jugal overlays the midsection of the surangular, obscuring much of its dorsal border (Fig 7). The articulation of the surangular with the angular is well defined, with a slight curve anteroventrally. There is small ridge that extends anteroposteriorly along the anteroventral margin of the surangular. The dorsal margin of surangular is visible through the orbit, above the jugal. There is a very steep rise to the paracoronoid process, with the jugal and postorbital obscuring the border and the muscle attachment point.

#### Angular

The lateral exposure of the angular is small, only covering the most posteroventral portion of the mandible (Figs 7 and 10). The dorsal margin curves anteroventrally and extends beneath the surangular.

#### Articular

Only the articular of the left side is visible. It is located behind the posterior margin of the surangular, with only its dorsal most margin visible (Fig 7). The margin is rounded with a rugose border, suggesting an attachment site for muscle or connective tissues (Fig 9).

#### Sclerotic ring

A partially articulated sclerotic ring is present within the orbit. It is composed of at least 14 trapezoidal plates. They are planar with a gently convex surface and crenulated internal margin, straight lateral margins and an external border with a subtle curvature (Fig 9). Each plate bears faint striations on the lateral surface.

Some of the ventral sclerotic plates have been crushed against the underlying palatal bones. Average length of a single plate is 17.7 mm, measured from the internal to external margins. The internal margins form a circle of approximately 42 mm diameter while the external margins define a circle of approximately 98 mm (Fig 9).

#### Palatal bones

Several bones of the palate are partially visible in the orbit, including the left pterygoid, both palatines, the parasphenoid portion of parabasisphenoid and both vomers. These are mostly obscured by the sclerotic ring (Fig 9). One vomer has been pushed through the external naris during compaction (Fig 8).

#### Dentition

**Fig 12:**
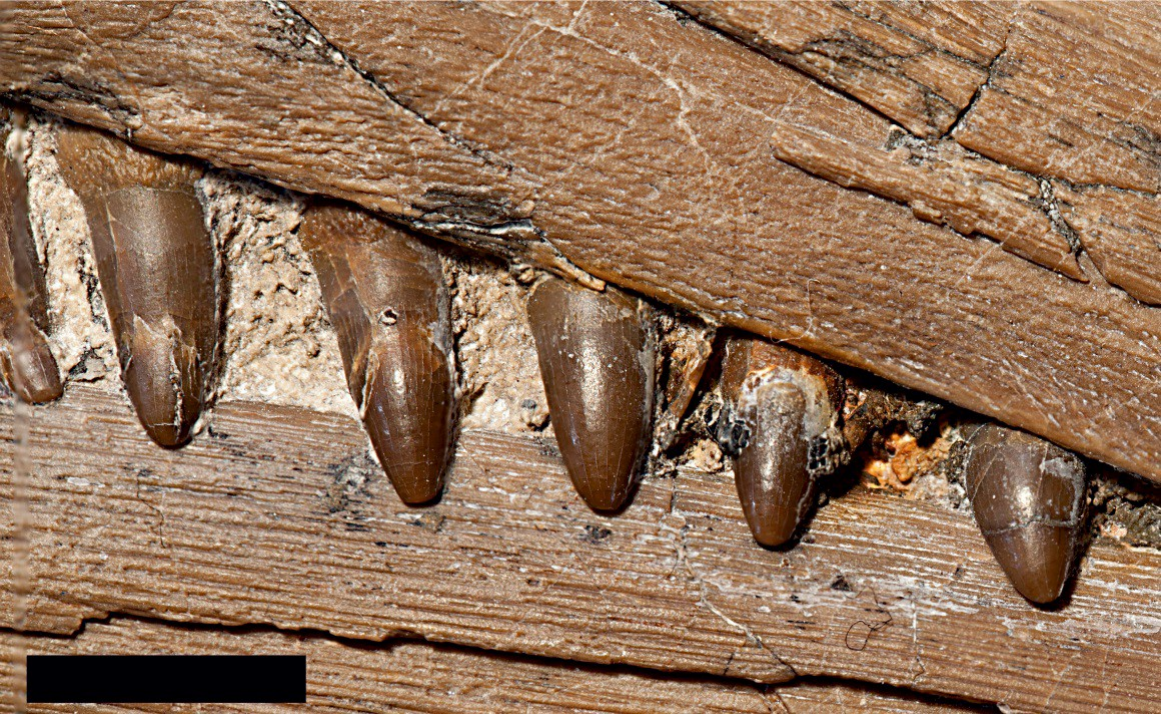
Dentition of *Thalassodraco etchesi* MJML K 1885, showing smooth enamel. Scale bar 10 mm. Photograph used by permission of the Etches Collection under the CC BY 40 license, original copyright 2016.

Teeth of the dentary, premaxilla and maxilla are similar, simple and extremely slender conical crowns on slightly more expanded roots. The dentition is isodont. The maximum tooth crown height, located in the rostrum is approximately 7 mm, giving a tooth length index of 0.2 (10 x maximum crown length of longest tooth/jaw length: *sensu* Motani [54]), but taking into account missing jaw length, with a conservative estimate of 50 mm, a more probable tooth length index of 0.19 is likely. The tooth crowns are slender, conical and slightly curved posteriorly with smooth enamel. The teeth are longer and thinner in the anterior portion of the jaw, becoming slightly shorter and wider posteriorly, with a maximum crown height of 7 mm and a minimum height of 4 mm (Fig 12). The base of the enamel layer and its contact with the root is well defined. The posterior most tooth crowns are shorter, with rounded tips. The slightly inflated roots bear fine vertical striations. Tooth morphology is not generally considered to be a useful taxonomic trait in Ichthyosauria [2, 6, 57–59] and possible ontogenetic differences in tooth form not been investigated. However, the teeth preserved in *Thalassodraco etchesi* are significantly distinct from any previously described ichthyosaur, is therefore considered here to be autapomorphic.

#### Vertebral column

There are thirty-three vertebrae preserved in the holotype of *Thalassodraco etchesi*. The first ten centra remain articulated, including the atlas-axis. There are 32 neural arches altogether, 20 of which are articulated on the main slab (Fig 4), articulated on the second slab (Fig 5A) and articulated on the third slab (Fig 5B). None of the neural arches are fused to the centra. The neural arches on the main block have the neural spine broken on the dorsal border of the anterior zygopophysis. Some have been slightly displaced by a few millimetres, but most remain articulated. No chevrons are preserved.

**Fig 13:**
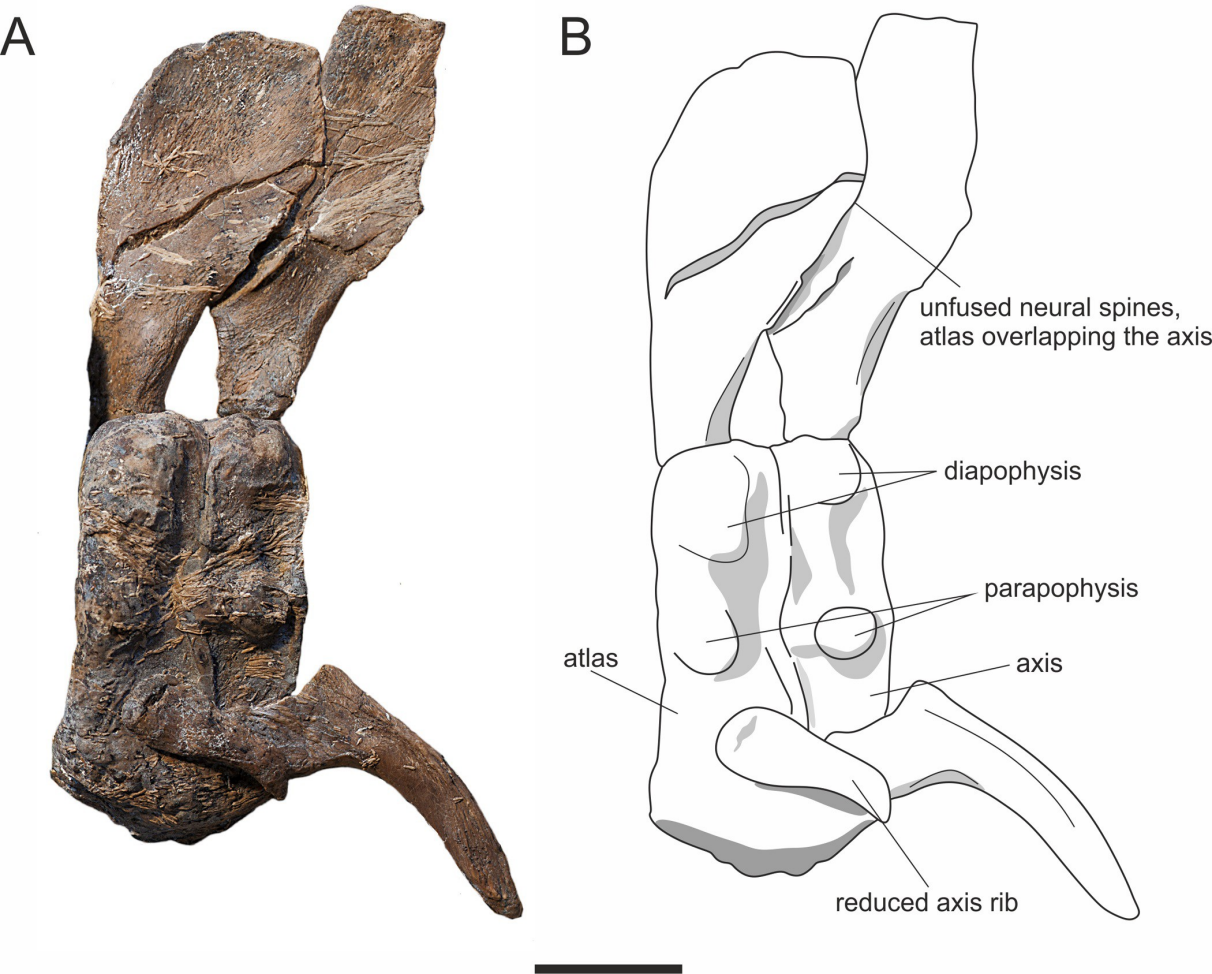
Atlas axis of *Thalassodraco etchesi*, MJML K 1885. A, photograph of the atlas axis. B, labelled interpretive drawing of the atlas axis. Scale bar 20 mm.

The atlas–axis complex is completely fused with a faint suture present dorsally (Fig 13). The diapophyses on both the atlas and axis are unpronounced compared to the sequential centra. The parapophysis on the atlas is small and shallow, whereas the parapophysis on the axis is larger and raised. A small, rudimentary rib articulates with the atlas parapophysis and lacks a tuberculum. A larger rib articulates with the axis but also lacks a prominent tuberculum. The axial intercentrum is absent. The neural spines of the atlas-axis are unfused. The atlas neural spine is tall and wide, with a flared anterior margin overlying the axis neural spine. The neural spine of the axis is tall and has a slight inflation of its posterior margin.

The first 8 centra from the atlas axis are articulated with their neural arches, but not fused (the normal condition in Ichthyosauria). The latter 8 are disarticulated. On the isolated slabs, there are 16 centra preserved, 13 complete and 2 partial, and one obscured by the rib cage (Fig 4). The neural spine height increases from 58 mm to 70 mm over the first 8 centra (Fig 4). Measurements of vertebrae are presented in S2 Table.

**Fig 14:**
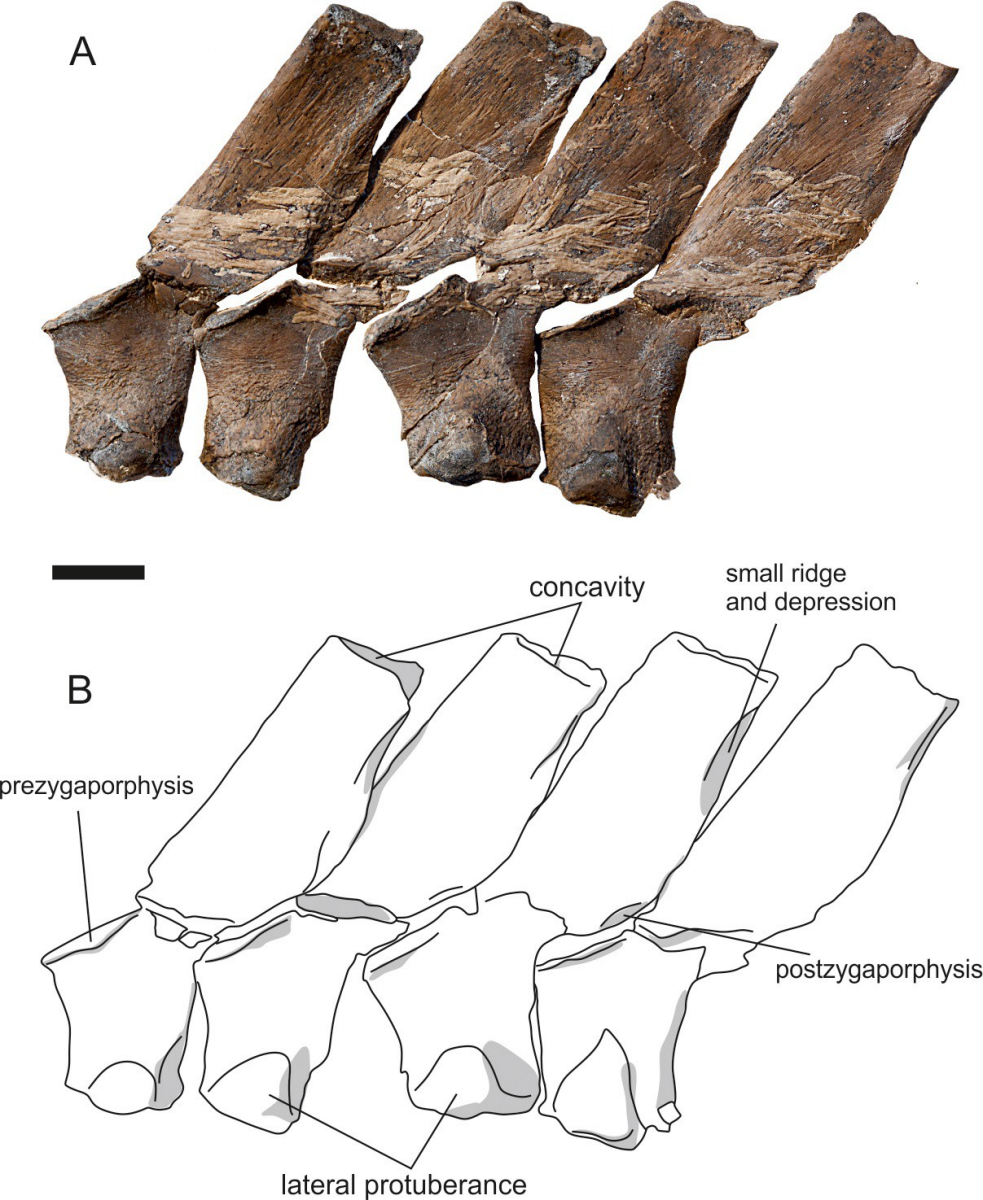
Neural spines of *Thalassodraco etchesi*, MJML K 1885. A, photograph of the neural spines. B, interpretive line drawing of the neural spines, highlighting the lateral protuberance on the neural arch. Scale bar 10 mm.

Twenty neural arches are preserved and articulated on the main specimen, including the atlas-axis complex (Figs 5 and 14). They increase in height from the 3^rd^ neural spine at 57 mm to the 20^th^ at 70 mm. Maximum height cannot be inferred due to disarticulation from neural spine 20. The first 14 neural spines lack the ‘V’ shaped apical notch on the dorsal margin of the neural spinesbut a slight notch is present in the last 6 articulated neural spines on the main slab (Fig 14B). The neural spines on the isolated slabs (Fig 5) lack the ‘V’ shaped notch. In dorsal view, the neural spine bears a concavity in its apex (Fig 14A and 14B). Extending dorsoventrally on the lateral surface of the neural spine is a low, oblique ridge, with a gentle depression posterior to the ridge sloping towards the posterior margin of the spine (Fig 11).

There is a prominent rim on the lateral border of the articulatory surface of the prezygopophyses, and a diapophyseal contribution of the neural arch to rib articulation on the ventral portion of the neural arch, which enlarges posteriorly (Fig 14), until neural arch 18 where it reduces. The diapophyseal contribution is also reduced on the neural arches present on the isolated slabs (Fig 5).

#### Rib cage

*Dorsal ribs*. On the main slab, there are 15 preserved articulated left dorsal ribs including and two cervical ribs articulating with the atlas-axis complex, 4 articulated right dorsal ribs and 3 disarticulated ribs. There are 12 dorsal ribs on the isolated slabs (Fig 5A–5D). The vertebral column has been displaced dorsally during burial, such that the ribs no longer are in contact with the lateral processes of the vertebral centra. Ribs with little to no curvature preserved on slab B (Fig 5B) are most likely from the posterior thorax or caudal peduncle. The cervical ribs are short, 20mm and 40mm in length respectively, with an oval cross section. The cervical ribs are single headed, with the second cervical rib bearing a prominent dorsal notch. The cross section of the ribs are T-shaped proximally, turning into 8-shaped then oval and rounded distally. The anterior dorsal ribs are deep, approximately 13 times as long as the height of the vertebral centra.

#### Gastralia

Many gastralia are preserved on all four slabs but are displaced and disarticulated. They are thin, slender, rod–like at one end and expand at the opposite end (Fig 5).

#### Appendicular skeleton

*Pectoral girdle*. The pectoral girdle is complete and articulated, with only a slight taphonomic offset of the interclavicle with the coracoids. The left scapula, distal end of the left clavicle and part of the anteromedial process of the right coracoid are obscured from full view by the skull and vertebral column respectively (see S3 Table for measurements). There is also a wide fracture that has been restored that occupies portions of the right clavicle, scapula and coracoid.

#### Clavicles

**Fig 15:**
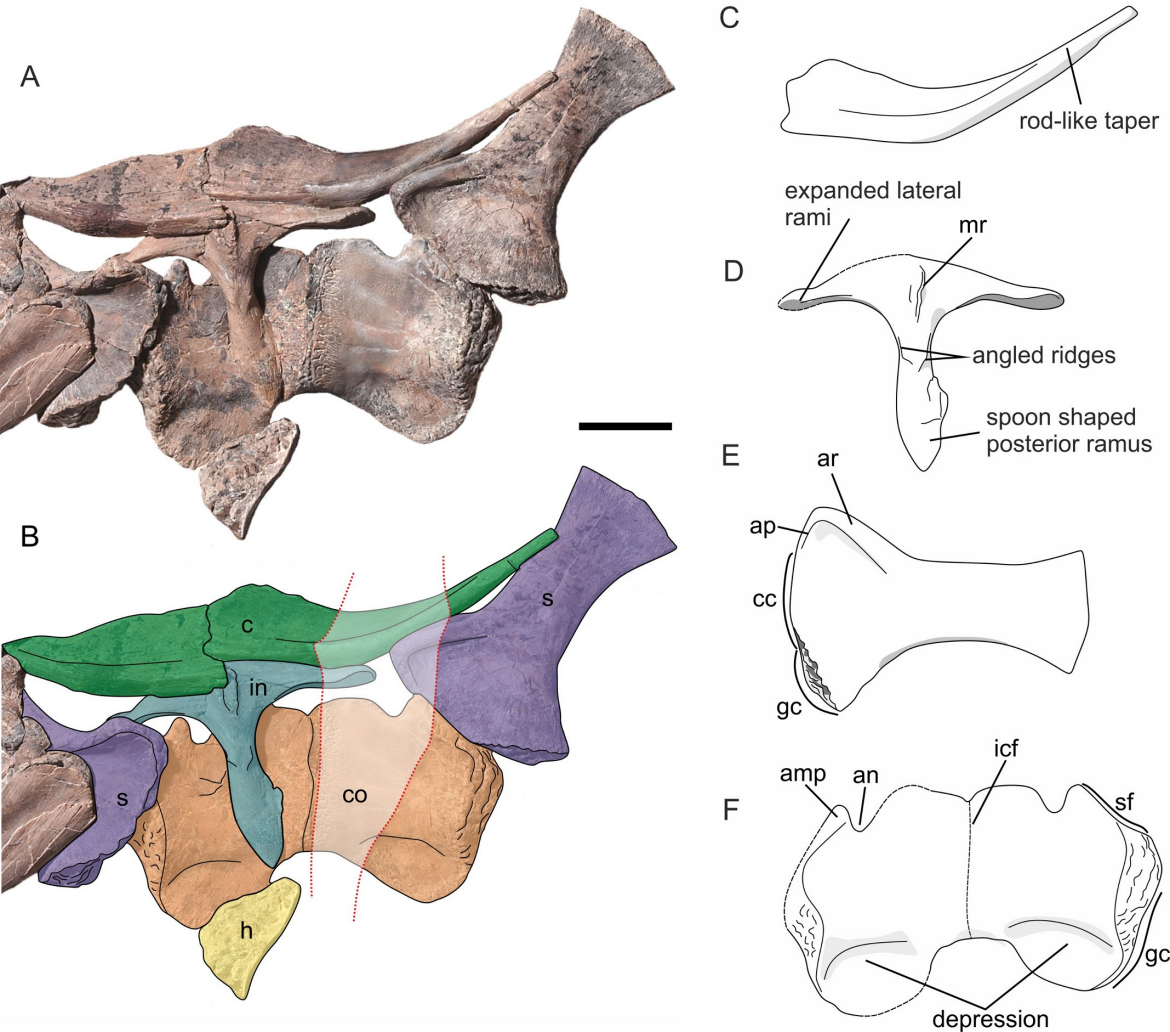
Pectoral girdle of *Thalassodraco etchesi*, MJML K1885. A, photograph of preserved pectoral girdle. B, line diagram of pectoral girdle. C, annotated line diagram of clavicle. D, annotated line diagram of interclavicle. E, annotated line diagram of right scapular. F, annotated line diagram of coracoids. For line diagrams, areas of restoration have been reconstructed from preserved portions of the pectoral girdle and personal observations. Abbreviations; amp, anteromedial process; an, anterior notch; ar, acromial ridge; ap, acromial process; c, clavicle; cc, coracoid contribution; co, coracoid; gc, glenoid contribution; h, humerus; icf, intercoracoid facet; in, interclavicle; mr, medial ridge; s, scapular; sf, scapular facet. Red dotted line indicates restored sections. Scale bar 100 mm.

Both clavicles are well preserved in anterior view. Medially the clavicles are flattened bones, tapering distally, and becoming rod-like, with a circular cross section. The dorsal margin expands medially around a third along its length (Fig 15A, 15B and 15C). The posterior border curves distally beyond its contact with the interclavicle. The scapular border subtends an angle of 147°.

#### Interclavicle

A T-shaped interclavicle is preserved in ventral aspect (Fig 15A, 15B and 15D). A medial ridge with a depression extends anteroposteriorly from the anterior most surface posteriorly along the medial line of the posterior ramus (Fig 15A and 15D). The ridged margins have a rugose texture. The lateral rami narrows dorsoventrally distally but expands anteroposteriorly distally, however the left lateral rami is partially reconstructed. The posterior ramus is spoon-shaped and bears two angled ridges directed medially (Fig 15A and 15D). It is longer than each of the lateral rami, but shorter than their combined lengths (see S3 Table for measurements).

#### Coracoids

Both coracoids are preserved in ventral view and in contact along the midline and the bone has a fibrous surface texture. There has been significant restoration to the right coracoid (Fig 15B). The left coracoid is subject to some crushing from the overlying interclavicle (Fig 15A and 15B). The coracoids are approximately the same length mediolaterally as anteroposteriorly wide. There are branching pit-like structures on the intercoracoid facet margin of the right coracoid, which are an artefact of restoration. The intercoracoidal facet is straight and there is a slight depression on the antero-ventral surface.

The scapular facet is slightly smaller than the glenoid facet. The scapular facet is offset by 120° to the glenoid contribution and facing anterolaterally and has a heavily pitted rugose texture (Fig 15F). The medial portion of the ventral margin extends distally into a protruding rounded margin.

The anteromedial process is prominent and narrow, forming the distal border of the anterior notch (Fig 15F). The anterior notch is relatively shallow in comparison with other Late Jurassic ophthalmosaurids, with a sloped medial border, and a steep distal border.

#### Scapula

Both scapulae are preserved, with the right scapula fully exposed in left lateral view (Fig 15A, 15B and 15E). Only the most proximal end of the right scapula is exposed. The right scapula comprises a wide and robust shaft, with curved lateral margins. The proximal blade is anteroposteriorly expanded and fan-like. The anterior portion of the scapula bears a prominent acromial ridge, with a broad concavity below. The distal blade is relatively narrower and modestly expanded anteroposteriorly. The distal blade forms two thirds of the total scapular length.

The articular surface of the proximal end of the scapular can be divided into three major portions, an anterior portion that supports the acromial process, and the glenoid and coracoid facets. The coracoid facet is larger than glenoid facet (Fig 15E). Glenoid facet is slightly thicker than the rest of the anterior margin. The two articular surfaces converge at an angle of 135°. The articular surface has a heavily pitted rugose texture, suggesting extensive cartilage attachment.

#### Forelimb

**Fig 16:**
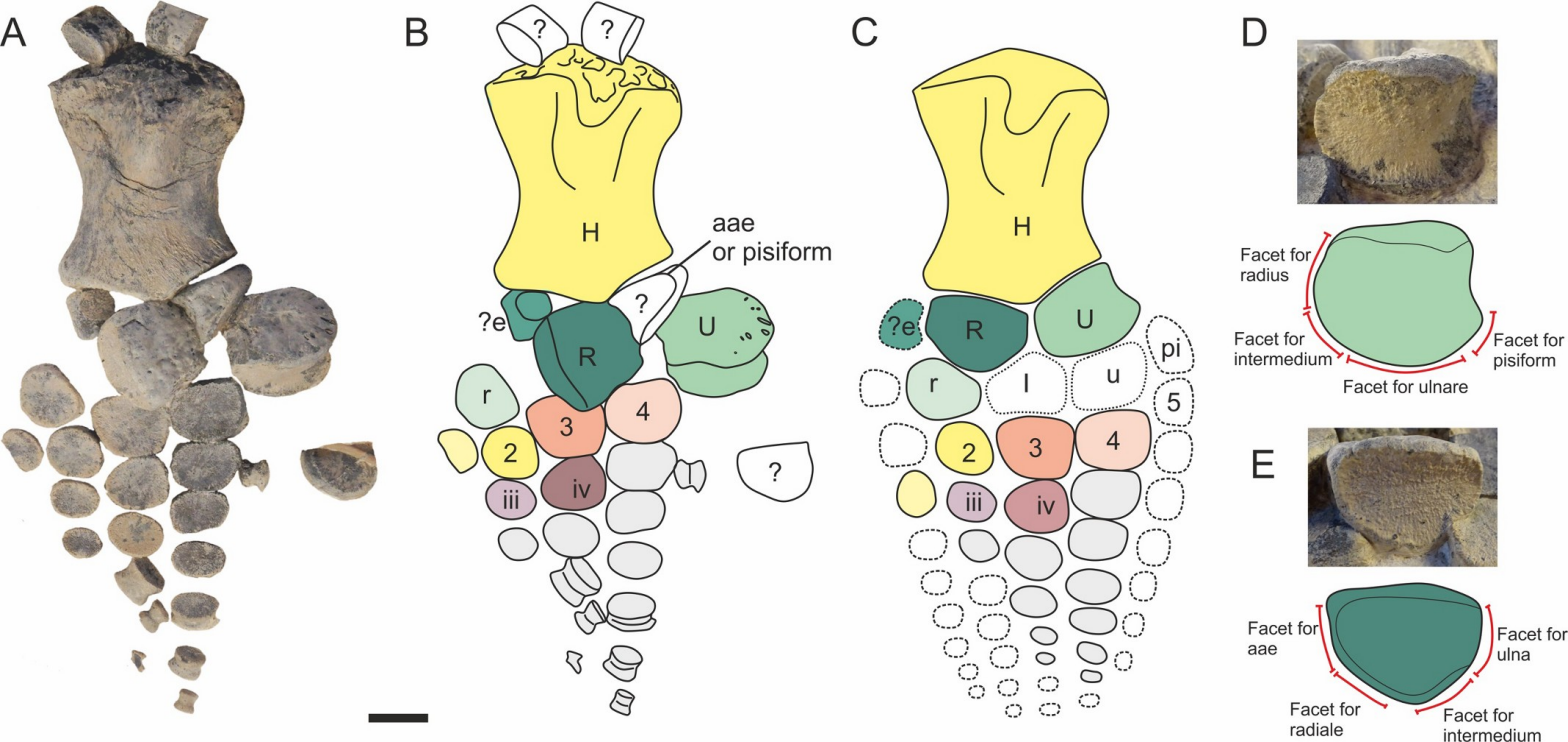
Forelimb of *Thalassodraco etchesi*, K1885. A, photograph of right forelimb. B, interpretation of forelimb. C, reconstruction of forelimb based on dorsal views of ulna and radius. D, interpretation of the humerus. E, dorsal surface of Ulna. F, dorsal surface of radius Abbreviations: dp, dorsal process; e, preaxial accessory element; ef, facet for preaxial accessory element; H, humerus; I, intermedium; pt, pitted texture; R, radius; r, radiale; rf, radial facet; U, ulna; u; ulnare; uf, ulnar facet. Scale bar 20 mm.

The left forelimb of MJML K1885 is slightly displaced from the pectoral girdle (Fig 16) and is displayed in dorsal view. The ulna, radius, intermedium and preaxial accessory elements are displaced, as are the most distal phalangeal elements. Some of the distal phalanges are missing. In general proportions, the forelimb appears to be relatively small compared to the overall body length (226 mm as preserved; 260 mm estimated length). In dorsal view, the forelimb is asymmetrical in respect to its long axis, being more broadly expanded preaxially. The postaxial margin is nearly straight, whereas the preaxial margin is slightly convex. Some of the distal preaxial elements have been displaced and some are missing (see S4 Table for measurements).

#### Humerus

The head of the right humerus lies ventral to the right coracoid (Fig 16). It is poorly preserved, and mostly concealed by matrix. Little useful morphological data can be extracted. The proximal and distal ends of the left humerus are nearly identical in maximum anteroposterior width. The proximal surface is convex and bears a prominent pitted texture for extensive cartilage attachment that would intervene between the humerus and glenoid facet. The dorsal trochanter is weakly developed as a low ridge extending anterodistally approximately one quarter of the way along the shaft. The shaft is slightly constricted at its midpoint, with a humeral length to midshaft width ratio of 1.9.

The distal surface is divided into subequal facets for ulna and radius and a considerably smaller facet anteriorly for a preaxial accessory element (Fig 16). Ulnar facet is deflected posterodistally, radial facet is directed distally and preaxial accessory facet directed anterodistally. The radial and ulnar facets subtend an angle of approximately 135°.

#### Zeugopodium and autopodium

Identity of digits here is based on criteria developed by Motani [54], and the arrangement of forelimb elements in other ophthalmosaurids. The epipodial row, which includes a radius, ulna and preaxial accessory element are displaced. The ulna and radius are identified by being the two largest elements, the ulna articulates with the radius and the ulnare, the latter is absent or concealed by matrix. The distal margin of the radius articulates with the preaxial accessory element, radiale, intermedium (absent) and ulna (Fig 16).

The ulna lacks a postaxial facet, indicating the absence of a pisiform. The posterior edge of the ulna has a slight concavity. The radius has facets for articulation with the preaxial element, ulna, intermedium, radiale and the preaxial element associated digit (Fig 16C and 16E). As the humerus lacks an articular facet for the intermedium, it is assumed that the two did not articulate. However, the intermedium is not preserved.

A possible preaxial element is presumed to contact the humerus, due to the presence of a small preaxial facet. In dorsal view, the preaxial element is tear-dropped shaped, however it has some damage to the anterodistal surface. The presence of a preaxial element is a synapomorphy of Ophthalmosauridae [58, 59]. Most of the leading edge and distal end of the forelimb is missing or disarticulated, so an associated preaxial digit is assumed in the restoration (Fig 16C), as this is seen in other ophthalmosaurids [54].

Preaxially to postaxially, the identity of the radiale and distal carpals 2, 3, 4 and 5 and absence of the intermedium and ulnare are easily established due to being in articulation, using their topology relative to the epipodial row. This interpretation recognises distal carpals 2 and 3 to lie distal to the radiale and distal carpal 4 to form the distal margin of the intermedium. Metacarpal 5 lies distal to the ulnare.

Digits 3, 4 and 5 are partially articulated, with some disarticulation of phalanges distally. The phalanges vary in shape. It is presumed there would have been four definite digits, identified as digits II, III, IV, V and possibly a preaxial digit. The proximal phalanges in digit V are subrectangular, but the phalanges present on other digits are oval or round. All digits reduce in size rapidly from the distal carpel to the most distal phalangeal element, indicating a relatively small forelimb. The referred specimen, MLMJ K1174 has a complete forelimb, which shows the same small forelimb morphology, and suggests very little is missing from the distal end of the forelimb of the holotype specimen.

### Pelvic girdle

#### Ischiopubis

**Fig 17:**
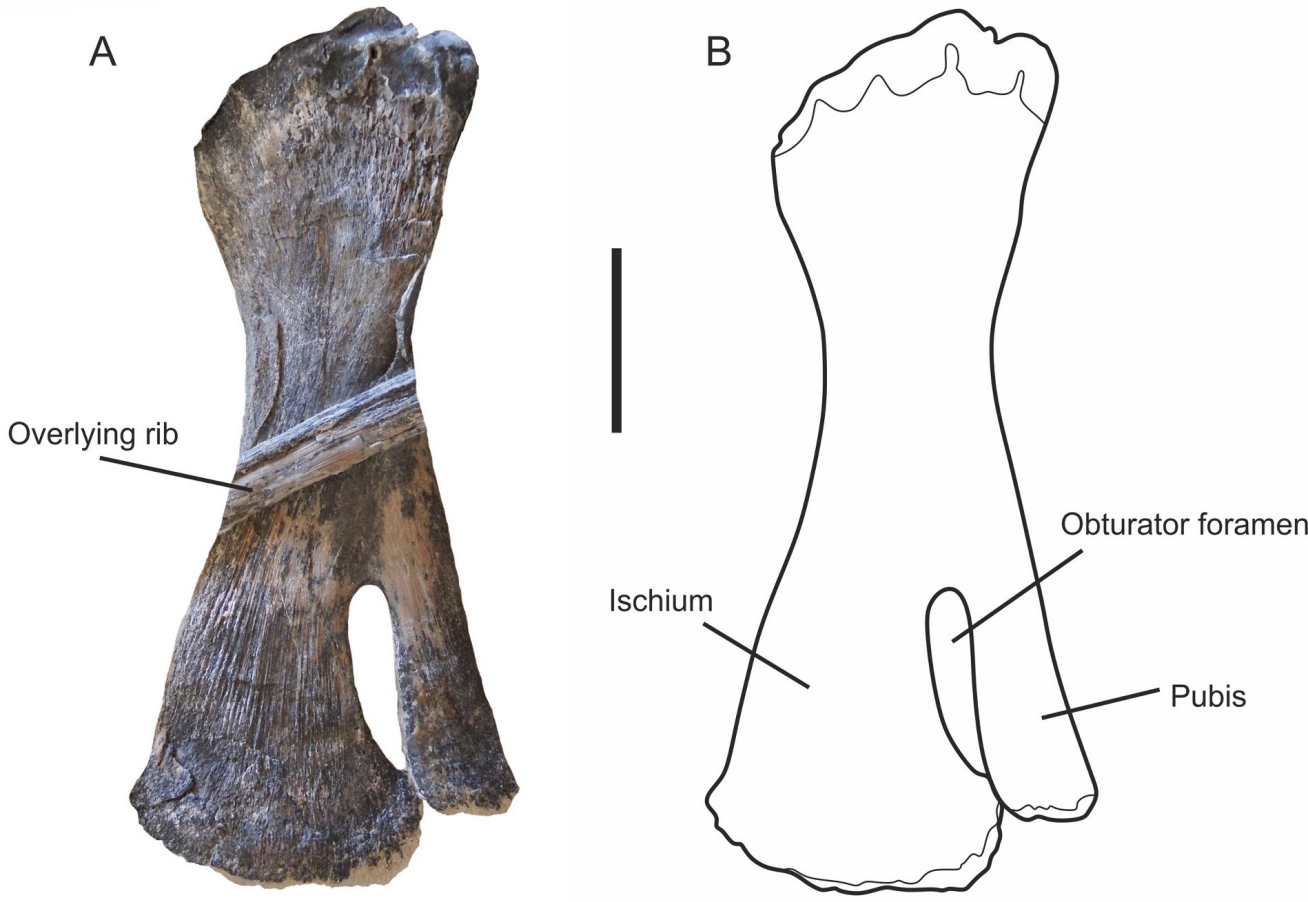
Ischiopubis of *Thalassodraco etchesi*, MJML K1885. A, photograph of isolated ischiopubis. B, line diagram of ischiopubis. Scale bar 20 mm.

The posterior portion of MJML K1885 is disarticulated, preserving only a complete ischiopubis on an isolated slab associated with 6 disarticulated vertebral centra, 2 ribs, 2 gastralia and 7 articulated neural spines (Fig 5B). Due to the disarticulated nature, it is unclear whether it is a left or right element. The ischium and pubis are fused proximally for approximately 0.75 of their length, with an elongate obturator foramen located closer to the medial margin, and the distal ends meeting, but not fused (Fig 17). The ischiopubis is uniformly flattened, with the ischium markedly wider than the pubis, with a flared distal margin. The pubis is thin, and rod-like (see S5 Table for measurements).

### Phylogenetic analysis

**Fig 18:**
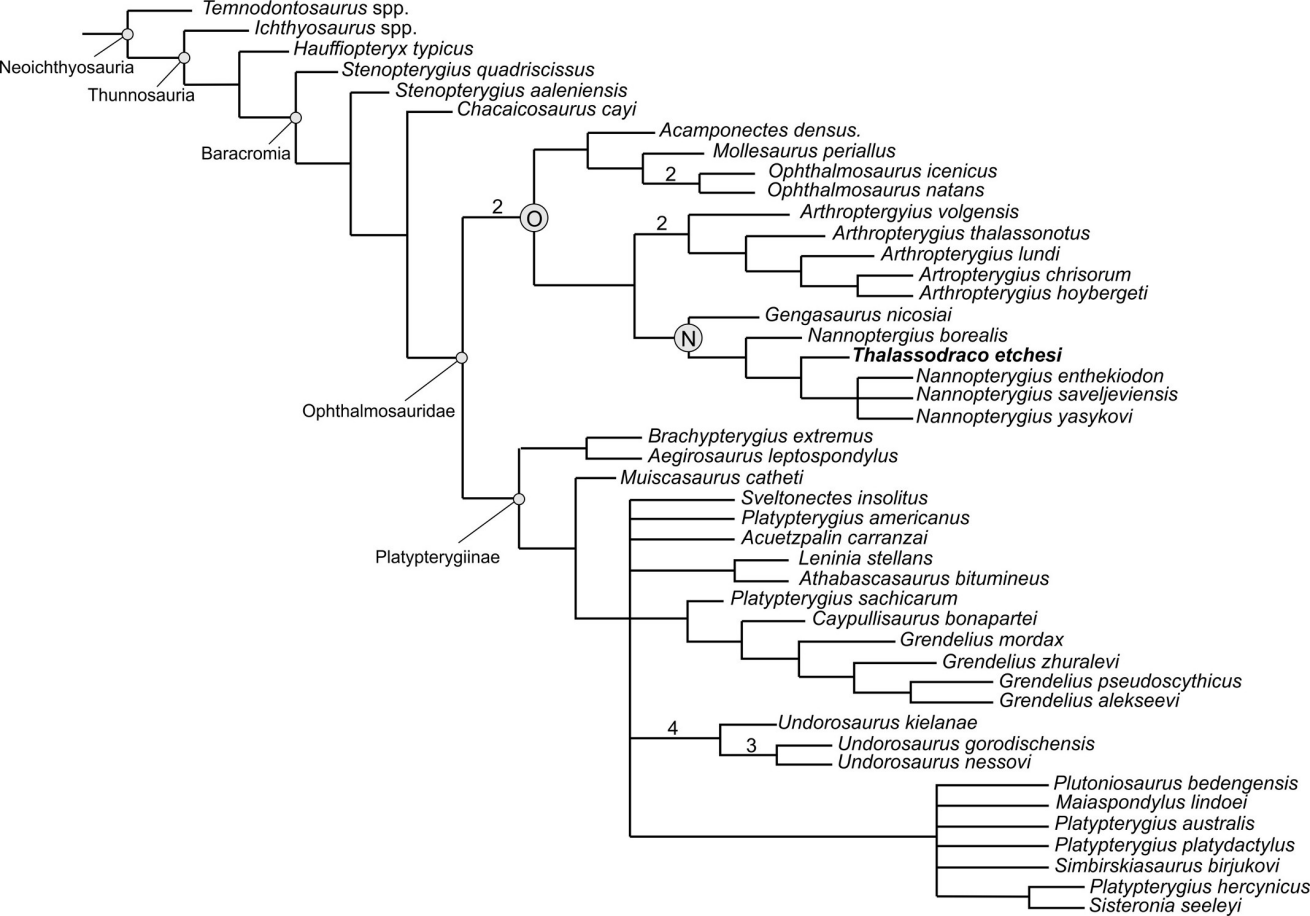
Phylogenetic position of *Thalassodraco etchesi*. A, a strict consensus tree recovered from the analysis. Bremer support values >1 are shown above the branches, bootstrap support values greater than 20 are shown below the branches. B, the clade that *Thalassodraco* falls into, showing synapomorphic characters. Abbreviations: N, *Nannopterygius* clade; O, Ophthalmosaurinae.

The phylogenetic analysis resulted in a strict consensus tree of 64 most parsimonious trees of 325 steps in length, with a consistency index (CI) of 0.397 and a retention index (RI) of 0.652 (Fig 18). The overall topology does not differ significantly from that of Zverkov & Jacobs [49].

The main topology of the analysis shows Ophthalmosauridae diverges into three clades; Ophthalmosaurinae and Platypterygiinae, and a third, *Nannopterygius* clade including *Nannopterygius* + *Thalassodraco* and *Gengasaurus*. This clade has been recovered in previous cladistic analyses by Zverkov & Efimov [60], Zverkov & Prilepskaya [61], and Zverkov & Jacobs [49].

The clade is poorly supported, with low Bremer values, probably due to the incomplete nature of the specimens, resulting in large gaps in the coding. Additionally, more complete material will produce a more resolved tree, giving a better understanding of the relationships of taxa in this clade.

Despite falling in the same clade there are considerable differences between *T*. *etchesi*, *Gengasaurus* [62] and *Nannopterygius* [49]. Notably *T*. *etchesi* has a wide postorbital bar of the jugal, whereas in *Nannopterygius*, this element is thin and gracile (char. 23); a jugal is not figured for the Italian ophthalmosaurid *Gengasaurus nicosiai* [62]. *T*. *etchesi* lacks a plate-like dorsal trochanter (char. 79); *T*. *etchesi* also lacks a prominent deltopectoral crest (char. 80); *T*. *etchesi* bears a facet on the humerus for an anterior accessory element, whereas *Nannopterygius enthekiodon* lacks this facet, *Gengasaurus* bears a facet for a preaxial element, however it is more pronounced than in *T*. *etchesi* (char. 82); *T*. *etchesi* has a large glenoid contribution on the scapular, but in *Nannopterygius* this is reduced (char. 73); *T*. *etchesi* has a prominent anterior notch on the coracoid, whereas *Nannopterygius* has a reduced notch (char. 70); *Nannopterygius* has a elongate coracoid while in *T*. *etchesi* it is somewhat shorter and wider (char. 69). These differences clearly warrant *Thalassodraco* placed in a distinct genus, albeit closely allied to *Nannopterygius* and *Gengasaurus*.

### Taphonomy

The holotype of *Thalassodraco etchesi* MJML K1885 is an incomplete, partially articulated skeleton collected on four large slabs (Figs 4 and 5) from the foreshore. Consequently, parts are missing or eroded. The anterior portion of the skeleton, including the skull, pectoral girdle, forelimb and dorsal vertebrae and ribs are articulated. Portions of the posterior skeleton appear to be disarticulated, and some elements widely separated from their skeletal counterparts. The specimen has been prepared from the underside, and this reveals the left lateral aspect of the skeleton.

The skull is partially compressed possibly due to collapsing during decay, sediment compaction or a combination of both, as suggested by numerous mid bone fractures. All the teeth are retained within the jaws and mostly remain in alignment within the tooth grooves. The sclerotic ring lies within the orbit and remains articulated but is crushed against overlying bones (Fig 9).

The first 9 vertebral centra, and 21 neural spines remain in articulation (Fig 4). The remaining vertebral centra and neural spines are scattered across the bedding plane (Fig 6). The articulated neural spines on the anterior portion of the skeleton display a conspicuous fracture extending through several arches from the pre to postzygoporhysis. Neural spines on the isolated slabs are not broken. Neural arches 20 and 21 have fractured due to the presence of underlying centra.

The first 17 left dorsal ribs remain associated but have all moved ventrally, so are no longer remain inarticulation with their centra. Four right dorsal ribs, posteriorly located, remain associated with their centra, but have been ‘flipped’ dorsally. Overlying disarticulated bones have caused some ribs to break due to compaction. The gastralia have been displaced and the gastral basket now underlying the ribs.

The pectoral girdle is articulated but has been directed ventrally during compaction. The right coracoid has some slight crushing caused by the more robust underlying right ramus of the interclavicle. The ulna, radius and intermedium are disarticulated as are the distal digits.

**Fig 19:**
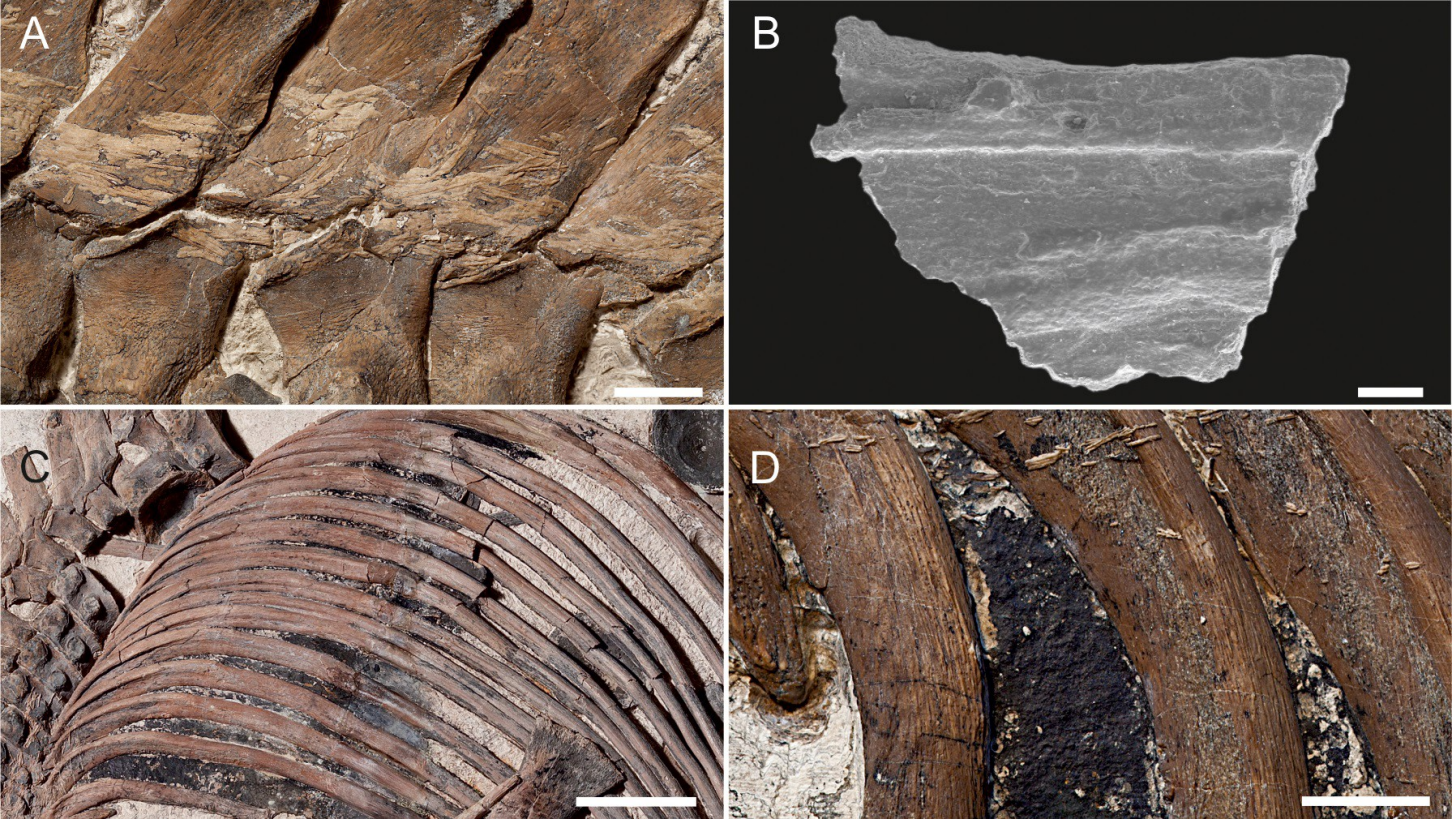
Summary of the taphonomic history of *Thalassodraco etchesi*, MJML K 1885. A, B, the carcass drifts in the water column, with the heavier head directed to the seafloor. C, the heavier skull arrives on the seafloor, with the skull penetrating the soft sediment, until it hits the harder, dewatered sediment below. D, the carcass starts to collapse laterally. If the gut and lungs are filled with gas, the posterior portion will remain buoyant, remaining out of the sediment. E, the carcass collapses and decomposes. The portion within the sediment remains articulated, whereas the posterior and right lateral portions are exposed to scavenging and scattering of bones through current action. Arrows indicate later compaction of the specimen as the sediment dewaters. Modified from Martill [65, 66].

In general, the bone appears to be well preserved with no cemented epifauna (note: many Kimmeridge Clay Fm. vertebrates have been colonised by serpulid worms). The lack of epifauna is possibly due a dysoxic sea floor environment [63, 64]. There are no signs of extreme compaction, like that seen in other Black Shale Lagerstätten (e.g. Posidonienschiefer examples of *Stenopteygius* [65]). All the bones retain their original shape, with only very slight distortion. An amorphous black material preserved in the body cavity with small, indeterminate clasts preserved within, is presumed to be decayed internal organs and perhaps stomach contents. Mineralised fibres, likely representing ossified ligaments are present on the anterior portion of the vertebral column and ribs (Fig 19), but it is unclear if this is in-vivo ossification or a diagenetic effect (soft tissue preservation). If the former applies, this may suggest the specimen represents a mature or even old adult with ossified ligaments.

**Fig 20:**
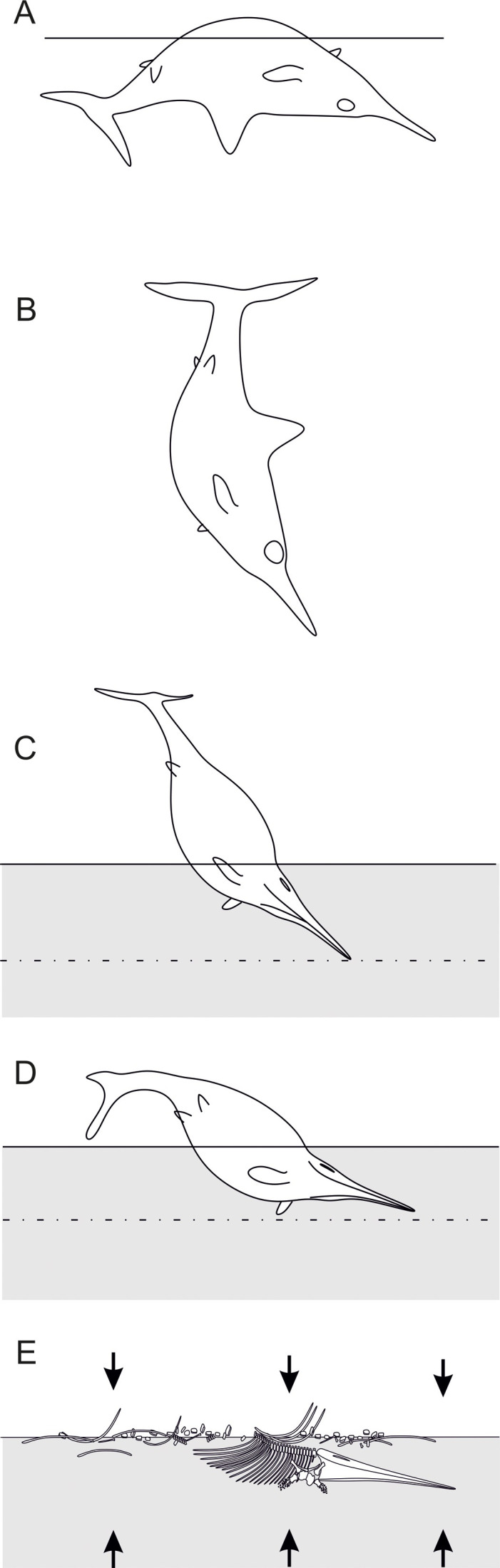
Ossified ligaments and decayed internal organs of *Thalassodraco etchesi*, MJML K 1885. A, ossified ligaments across the neural spines. B, SEM image of the ossified ligament. C, black material, presumed decayed internal organs, in the upper body cavity. D, magnified view of the black material in the body cavity. Scale bar in A, D represents 10 mm, B represents 200 μm and C, 100 mm. Photographs A, C, D used by permission of the Etches Collection under the CC BY 40 license, original copyright 2016.

The specimen appears to have come to rest on the seafloor on its left anterior lateral side (Fig 20), which possibly sank into a soft, coccolith-rich substrate, allowing for this portion of the specimen to be better preserved and remain articulated. The posterior portion of the animal was likely left exposed on the seafloor, available to scavengers and perhaps current action, where disarticulation allowed the scattering of bones across the seafloor.

## Discussion

### Comparisons

*Thalassodraco etchesi* possesses several features that distinguish it from other ichthyosaurs, whilst sharing a number of similarities with other Late Jurassic and Early Cretaceous ichthyosaur taxa. These are discussed below.

*T*. *etchesi* possesses a elongate supranarial process of the premaxillae, contacting the external naris, which is a character shared with *Brachypterygius* [19], *Caypullisaurus* [67–69], *Nannopterygius* [49], *Aegirosaurus* [55, 70] and but is not present in *Sveltonectes* [71]. In *Ophthalmosaurus* [18], *Arthropterygius* [61, 72] and *Undorosaurus* [60, 73] the supranarial process is reduced. The supranarial process is not preserved in *Gengasaurus* [68].

The subnarial process contacts the jugal in *T*. *etchesi*, a trait shared with *Brachypterygius*, *Undorosaurus*, *Nannopterygius* and *Arthropterygius*. The subnarial process does not contact the jugal in *Ophthalmosaurus*, *Sveltonectes*, *Aegirosaurus* or, *Caypullisaurus *[18, 19, 53, 57, 58, 63–71, 73].

Prefrontal of *T*. *etchesi* contacts the external naris, also present in *Aegirosaurus*, *Sveltonectes*, *Ophthalmosaurus* and *Nannopterygius*. The prefrontal does not contact the external naris in *Brachypterygius*, *Caypullisaurus*, *Undorosaurus*, *Grendelius* or *Arthropterygius* [18, 19, 49, 57, 58, 60, 61, 63, 70, 72, 73].

In *T*. *etchesi* the postorbital is wide and robust, with a straight anteroventral margin, with sharp curve dorsally. In *Sveltonectes* and *Nannoptergius*, the postorbital is not as wide, with a curved anterior and posterior margin, while in *Caypullisaurus* the postorbital is wider than *T*. *etchesi*. *Undorosaurus* has an angular anterior margin and is wide. The postorbital is thin in *Aegirosaurus*, *Ophthalmosaurus* and *Arthropterygius*.

**Fig 21:**
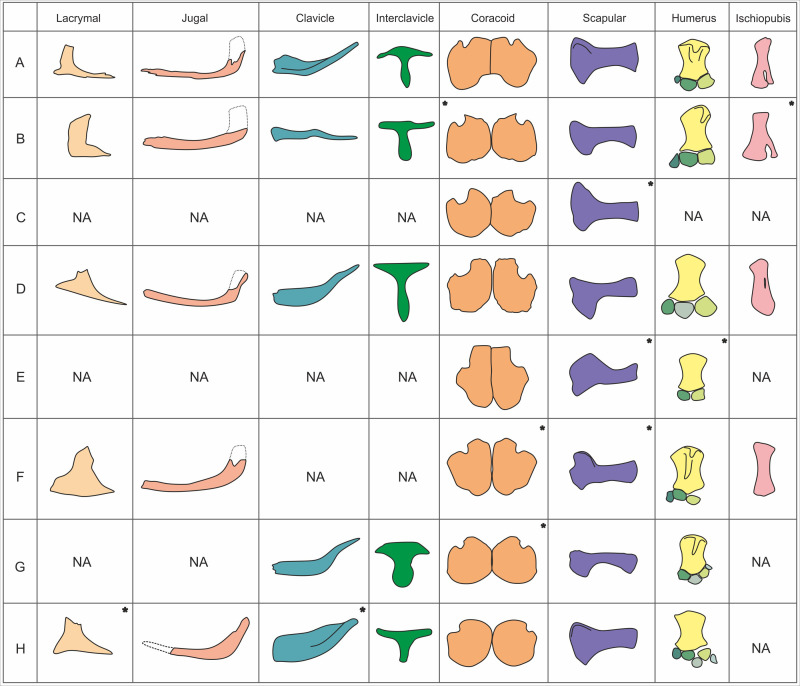
Comparisons with other Late Jurassic and Early Cretaceous ichthyosaurs. A, *Thalassodraco etchesi*, MJML K1885, B, *Undorosaurus gorodischensis*, PMO 214. 578, C, *Acamptonectes densus*, GLAHM 132588, D, *Ophthalmosaurus icenicus*, NHMUK PV R2137, E, *Nannopterygius enthekiodon*, NHMUK PV 46497, F, *Sveltonectes insolitus*, IRSNB R269, G, *Grendelius alekseevi*, YKM 56702, H, *Arthropterygius chrisorm*, jugal, clavicle and scapular from specimen CCMGE 17-44/13328, interclavicle from specimen SGM 1573, coracoids and scapula from specimen CCMGE 3-16/13328, lacrymal from *A*. *lundi* PMO 222.654. ‘*’ indicating elements that have been mirrored for comparative purposes. Not to scale.

The posterior border of the lachrymal of *T*. *etchesi* is steeply curved (Fig 21), but is only slightly curved in *Ophthalmosaurus*, *Sveltonectes*, *Arthropterygius*, *Nannopterygius*, *Aegirosaurus* and *Caypullisaurus*. In *Undorosaurus* and *Brachypterygius* the posterior border is sharply curved through 90 degrees.

The lachrymal contacts the external naris in *T*. *etchesi*, a character shared with *Ophthalmosaurus*, *Caypullisaurus*, *Aegirosaurus*, *Sveltonectes*, *Nannopterygius*, *Brachypterygius* and *Arthropterygius*, but in *Undorosaurus* the lachrymal does not contact the external naris.

The jugal of *T*. *etchesi* is straight with a posterior dorsal upturn and an elongated dorsal process (Fig 21). This condition is also present in *Ophthalmosaurus*, *Nannopterygius* and *Arthropterygius*. In *Undorosaurus* and *Brachypterygius* the jugal is straight along its entirety. *Caypullisaurus* has a straight jugal with a slight upturn posteriorly, while *Aegirosaurus* and *Brachypterygius* have a curved jugal with no posterior upturn.

The jugal of *T*. *etchesi* contacts the premaxilla, a character shared with *Brachypterygius*, *Nannopterygius* and *Arthropterygius*. However, in *Aegirosaurus*, *Caypullisaurus*, *Ophthalmosaurus*, *Undorosaurus* and *Sveltonectes* the jugal does not contact the premaxilla.

The jugal articulates exclusively with the postorbital in lateral view in *T*. *etchesi*, which is also seen in *Sveltonectes* and *Arthropterygius*. The jugal does articulate with the postorbital and quadratojugal in *Brachypterygius*, *Aegirosaurus*, *Caypullisaurus*, *Ophthalmosaurus* and *Undorosaurus*

The angular of *T*. *etchesi* has a small lateral exposure, which is shared with *Sveltonectes* and *Aegirosaurus*. *Ophthalmosaurus*, *Brachypterygius*, *Nannopterygius* and *Arthropterygius* have a considerably larger lateral exposure of the angular. In *Caypullisaurus* and *Undorosaurus* the exposure is larger still.

The lateral exposure of the maxilla is short in *T*. *etchesi*, which is also present in, *Aegirosaurus*, *Ophthalmosaurus* and *Nannopterygius*. In *Sveltonectes*, the maxilla exposure is greatly reduced. The maxilla lateral exposure is extensive in *Caypullisaurus* and *Undorosaurus*. In *Brachypterygius*/*Grendelius* and *Arthropterygius lundi* the exposure of the maxilla is anteroposteriorly long, but short dorsoventrally.

The teeth of *T*. *etchesi* are small, delicate and bear smooth surface enamel, with approximately 70 teeth preserved in the upper tooth row. This dentition is most similar to that of *Aegirosaurus*, which has delicate teeth with enamel that bears minute ridges or are completely smooth and 60–65 teeth in the upper tooth row. *Nannopterygius* also bears small teeth with minute ridges to smooth enamel [49, 74, 75]. Most other Late Jurassic and Early Cretaceous ichthyosaurs, including *Undorosaurus*, *Acamponectes* and *Brachypterygius* have large, robust teeth with fine enamel ridges. *Undorosaurus* and *Brachypterygius* have 53 teeth in the upper tooth row. In *Acamptonectes densus* Fischer et al. [76], the teeth are faintly straited, but only on the basal two-thirds [76]. In *Arthropterygius hoybergeti* Druckenmiller et al. [77], the teeth are large and striated [61], where as in *Arthropterygius lundi* Roberts et al. [78], the teeth are not figured, but described as diminutive (9 mm tall) and having fine ridges on all sides [78]. In *Sveltonectes insolitus* Fischer et al. [71], the teeth are up to 19 mm in total height and the crowns have delicate longitudinal striations. In *Maiaspondylus lindoei* Maxwell and Caldwell [79], the tooth crowns are smooth as in *T*. *etchesi* but are more robust in *M*. *lindoei*. In *Muiscasaurus catheti* Maxwell et al. [80], the teeth are also smooth, but unlike *T*. *etchesi*, they are sharply pointed and wide at the crown base [80]. In the Kimmeridgian *Nannopterygius enthekiodon* Hulke [75], the teeth are unknown in the holotype, but in a referred specimen (NHMUK uncatalogued Zverkov and Jacobs [49], Fig 2) the teeth are smooth, slender, with bulbous roots and sharp crown apices. However, it is not certain this specimen can be referred to *Nannopterygius*.

The clavicle of *T*. *etchesi* has a distally expanded clavicle plate, extending for over half the overall length of the clavicle, with a rod-like distal portion (Fig 21). A similar condition is seen in *Ophthalmosaurus* and *Grendelius* [81, 82] (Fig 21). However, the rod-like distal portion is curved in *Ophthalmosaurus* and *Grendelius*, and straight in *T*. *etchesi*. In *Undorosaurus* the entire clavicle is rod-like. The clavicle plate is not set as medially high in *Grendelius* and is set very high medially, extending along the length of the clavicle in *Arthropterygius*.

The interclavicle of *T*. *etches* is T-shaped with a spoon shaped posterior ramus with a medial ridge on the anterior portion of posterior ramus. The lateral rami narrow dorsoventrally distally, flaring out anteroposteriorly at the distal most portion. A similar condition is seen in *Ophthalmosaurus*, *Aegirosaurus* and *Caypullisaurus* [18, 55, 69], however the latter has wider lateral rami (Fig 21). *Arthropterygius* has a robust, T-shaped interclavicle with a bulge in the middle of the posterior ramus [63]. *Nannopterygius* and *Grendelius* [49, 82] have a wide medial and posterior ramus and a medial ridge extending laterally across the medial ramus, with a rounded protuberance in the middle of the two lateral rami. *Undorosaurus* interclavicle has a rounded protuberance in the middle of the two lateral rami, and the posterior ramus flares posteriorly into a wide spoon-shape, significantly more so than in *Nannopterygius* and *Grendelius* (Fig 21). In *Gengasaurus*, the transverse bar is diamond shaped, and thickened along the anterior margin. The posterior ramus is not preserved.

The anteromedial process of the coracoid is poorly pronounced and rounded in *T*. *etchesi*, similar to that of *Sveltonectes* and *Grendelius*. In *Acamptonectes*, *Ophthalmosaurus* and *Nannopterygius* the process is pronounced and protrudes anteriorly more than in any other ichthyosaur (Fig 21).

The anterior notch of the coracoid in *T*. *etchesi* is narrow, similar to the condition in *Undorosaurus*. However, the distal margin of the anterior notch is straight and directed anteriorly. The anterior notch is wider in *Undorosaurus* than in *T*. *etches* (Fig 21). In *Sveltonectes* and *Grendelius* the distal margin is slightly more laterally directed. In *Ophthalmosaurus*, *Nannopterygius* and *Arthropterygius*, the notch is wide with a laterally directed distal margin (Fig 21).

The intercoracoidal margins are straight in *T*. *etchesi*, a trait shared with *Ophthalmosaurus*, *Nannopterygius*, *Sveltonectes* and *Grendelius* (Fig 21).

The posterior margins of the coracoid are concave at the intercoracoidal margin, and flare out posterolaterally, creating a U-shaped posterior margin. This is considered an autapomorphy of *T*. *etchesi*. As all other ophthalmosaurids have convex posterior coracoid margins and lack the concavity at the intercoracoidal margin (Fig 21).

The scapula of *T*. *etchesi* bears a prominent acromion process, also present in *Arthropterygius* and *Ophthalmosaurus* (Fig 21). In *Grendelius* and *Undorosaurus* the acromion process is reduced, but in *Acamptonectes*, *Nannopterygius* and *Sveltonectes* this process is highly exaggerated (Fig 21) [18, 49, 61, 62, 71, 75, 82, 83].

The scapula shaft of *T*. *etchesi* is robust and straight, with an anterior and posterior expansion at the distal margin. In *Gengasaurus*, *Ophthalmosaurus* and *Sveltonectes*, the scapula has a slight anterior and posterior expansion of the distal margin, however the shafts are not as robust (Fig 21). *Acamptonectes* and *Arthropterygius* have a robust shaft but shows no expansion of the distal margin. *Undorosaurus* and *Grendelius* have a concave posterior margin but only shows posterior expansion of the distal margin. The anterior margin in *Nannopterygius* is concave, and the distal margin shows only anterior expansion (Fig 21).

The scapula has a prominent and anteroposteriorly thickened ventral portion of the acromial process, this is also present in most ophthalmosaurids while in *Sveltonectes* and *Arthropterygius* it is narrower still (Fig 21).

The dorsal trochanter of the humerus is weakly developed on *T*. *etchesi* and extends only 0.25 the way down the shaft. Whereas the dorsal trochanter is ‘plate-like’ in *Ophthalmosaurus*, *Brachypterygius*, *Sveltonectes*, *Grendelius*, and *Acamptonectes* and extends at least halfway down the shaft of the humerus (Fig 21). It is described as ‘tall and narrow’ for *Arthropterygius* by Roberts et al. [78]. In *Nannopterygius* a dorsal trochanter extends only 0.25 to 0.5 down the shaft of the humerus but is plate-like (Fig 21).

The dorsal trochanter is medially placed in *T*. *etchesi*, a condition also seen in *Nannopterygius* and *Arthropterygius*. It is posteriorly placed in *Undorosaurus*, *Sveltonectes*, and *Grendelius* and *Brachypterygius* (Fig 21).

The ventral process of the humerus is reduced to a gentle curve, with no substantial protuberance in *T*. *etchesi*, unlike *Ophthalmosaurus*, *Brachypterygius* and *Nannopterygius* which have a prominent ventral process.

In *T*. *etchesi*, the intermedium does not contact the humerus, a character also shared with *Nannopterygius*, *Sveltonectes*, *Arthropterygius*, *Gengasaurus* and *Undorosaurus*. The intermedium does contact the humerus in *Brachypterygius*, *Aegirosaurus* and *Grendelius*.

Distal phalanges are rounded in *T*. *etchesi*, also present in *Arthropterygius*, *Gengasaurus*, *Ophthalmosaurus* and *Nannopterygius*. The distal phalanges are rectangular in *Brachypterygius*, *Aegirosaurus*, *Caypullisaurus*, *Sveltonectes* and *Grendelius*.

The ulna of *T*. *etchesi* has concave posterior surface, a character shared with *Acamptonectes*, *Ophthalmosaurus* and *Nannopterygius* (Fig 21). The posterior surface of the ulna is either convex or straight in *Brachypterygius*, *Aegirosaurus*, *Caypullisaurus*, *Undorosaurus*, *Grendelius* and *Arthropterygius* (Fig 21).

The ischiopubis of *T*. *etchesi* is fused only medially with an obturator foramen, with the distal portions contacting. The ischiopubis is fused only medially in *Undorosaurus*, however the distal portions do not contact (Fig 21). The ischiopubis in *Ophthalmosaurus* [11], is fused medially and distally with an obturator foramen. The whole ischiopubis is completely fused in *Aegirosaurus*, *Sveltonectes* and *Arthropterygius* and lack an obturator foramen (Fig 21). The ischiopubis is unfused in *Caypullisaurus* [69].

### Implications for Late Jurassic ichthyosaur diversity

The occurrence of the new genus of ichthyosaur increases the number of Upper Jurassic UK ichthyosaur genera to five; the previously known being *Ophthalmosaurus* Seeley [82], *Brachypterygius* von Huene [83], *Grendelius* McGowen [85], and *Nannopterygius* von Huene [84]. *Macropterygius* is now considered a nomen dubium by Zverkov & Jacobs [49], *contra* Moon and Kirton [19]. *Thalassodraco etchesi* also adds to the diversity of Kimmeridge Clay Formation ichthyosaurs, with only *Nannopterygius enthekiodon*, *Grendelius mordax* and *Brachypterygius extremus* previously described from this horizon. While some Kimmeridge Clay Formation genera have been recorded outside of the formation, uniquely, the ichthyosaur species of the Kimmeridge Clay Fm. are endemic. It is highly likely that Kimmeridgian ichthyosaur diversity will increase with the study of new and undescribed material in the Etches Collection.

Ichthyosaurs are known from several other Late Jurassic formations including in North America (Wyoming), Mexico, Europe, European Russia, Svalbard and Argentina, with only 5 genera becoming widespread; *Arthropterygius* Maxwell [86]; *Grendelius* McGowen [85]; *Nannopterygius* von Huene [84]; *Ophthalmosaurus* Seeley [82] and *Undorosaurus* Efimov [87], but these are not evenly distributed.

The two most widespread genera are *Arthropterygius*, occurring in Northern Canada, Svalbard, Franz Josef Land, European Russia and Argentina [59, 72, 78, 88, 89, 90–92] and *Ophthalmosaurus* occurring in the Callovian to Oxfordian of England, the Oxfordian of western USA (Wyoming) and the Tithonian of European Russia, Mexico and possibly Argentina [19, 93–95]. Two possible routes for the dispersal of these genera have been proposed: The ‘Boreal route’, a North-South directional dispersal extending from the Arctic Sea, along the Palaeopacific coast of the North American continent and northern Pangea into the Neuquen Basin of Argentina [96, 97] and so-called the ‘Hispanic corridor’, a largely epicontinental seaway allowing for the exchange of fauna between the Eastern Palaeopacfic and the Western Tethys basins between Gondwana and Eastern Laurasia [97–103]. Other ichthyosaur genera with a wide distribution appear to be restricted to the Northern Hemisphere at high latitudes, dispersing via small seaways located between the Artic Sea, West Siberian Sea, Middle Russian Sea, Polish Sea, Anglo-Paris Basin and the Palaeo-Atlantic Ocean [97].

A truly global distribution of ichthyosaur genera isn’t seen until the Early Cretaceous with *Platypterygius*, with 8 known species which occur widely across the northern and southern hemispheres [103–111]. However, this genus is in need of review, and possibly it will be found to be polyphyletic.

Due to the network of numerous seaways allowing for dispersal across Europe, Russia and the Artic during the Late Jurassic, the new genus *Thalassodraco*, would most likely disperse, at least into Europe. However, its occurrence so far within the UK only can easily be explained as sampling bias and perhaps misidentification of remains in equivalent deposits elsewhere.

## Conclusions

*Thalassodraco etchesi* gen. et sp. nov. is a new ophthalmosaurid ichthyosaur from the Late Jurassic Kimmeridge Clay of Dorset, UK. It pocesses a unique combination of cranial and postcranial features, including the autapomorphic features; a large rounded protuberance on the supratemporal bone; thin L-shaped lachrymal, with a steeply curved posterior border; ~70 teeth on the upper tooth row; T- shaped interclavicle, with a spoon shaped posterior ramus with a medial ridge on the anterior portion of posterior ramus. A phylogenetic analysis finds *Thalassodraco* nested within a clade within Ophthalmosauridae, with *Nannopterygius*, *Paraophthalmosaurus* and *Gengasaurus*. Although closely related *Nannopterygius*, it bears numerous notable differences, which warrant *T*. *etchesi* to be a separate genus.

*T*. *etchesi* adds to our knowledge of the diversity of ichthyosaurs in the Late Jurassic, increasing the number of ichthyosaurs from the Kimmeridge Clay to five. Knowledge of Kimmeridge Clay ichthyosaurs is far from complete, with many new undescribed taxa currently within the Etches Collection. The material from the Etches Collection will continue to expand our knowledge of the diversity, ecology and distribution of Late Jurassic ichthyosaurs.

## Supporting information

S1 File (XLSX)

S1 Table Selected cranial measurements (in mm). (DOCX)

S2 Table Selected axial measurements (in mm). (DOCX)

S3 Table Selected pectoral girdle measurements (in mm). (DOCX)

S4 Table Selected forelimb measurements (in mm). (DOCX)

S5 Table Selected pelvic girdle measurements (in mm). (DOCX)
