# Supplementary material for: A new ophthalmosaurid ichthyosaur from the Upper Jurassic (Early Tithonian) Kimmeridge Clay of Dorset, UK, with implications for Late Jurassic ichthyosaur diversity
Source: PLoS One. 2020 Dec 9;15(12):e0241700. doi: 10.1371/journal.pone.0241700 (PMC7725355; doi:10.1371/journal.pone.0241700)
Supplement: S1 Table — (DOCX) [file pone.0241700.s002.docx]

S1 Table. Selected cranial measurements (in mm).

| Maximum skull length | 520 |
| --- | --- |
| Anteroposterior length of orbit | 131 |
| Dorsoventral length of orbit | 85 |
| Preorbital (snout) length | 303 |
| Total length of tooth row | 234 |
| Anteroposterior length of postorbital bar | 32 |
