# Supplementary material for: A new ophthalmosaurid ichthyosaur from the Upper Jurassic (Early Tithonian) Kimmeridge Clay of Dorset, UK, with implications for Late Jurassic ichthyosaur diversity
Source: PLoS One. 2020 Dec 9;15(12):e0241700. doi: 10.1371/journal.pone.0241700 (PMC7725355; doi:10.1371/journal.pone.0241700)
Supplement: S2 Table — (DOCX) [file pone.0241700.s003.docx]

S2 Table. Selected axial measurements (in mm).

| **Centrum number** | **Height (visible)** | **Length** | **Neural arch height** |
| --- | --- | --- | --- |
| 1, 2 (Altas/axis) | 58.5 | 33 | 58 |
|  |  |  | 55 |
| 3 | 47.7 | 18.8 | 57 |
| 4 | 43.4 | 17.7 | 58 |
| 5 | 38.9 | 19.7 | 58 |
| 6 | 34.4 | 18 | 60 |
| 7 | 35 | 19.2 | 61 |
| 8 | 36.1 | 21 | 63 |
| 9 | 38.4 | 19.8 | 62 |
| 10 | 44.1 | 22.3 | 66 |
| 11 | - | - | 62 |
| 12 | - | - | 62 |
| 13 | - | - | 46 visible |
| 14 | - | - | 67 |
| 15 | - | - | 66 |
| 16 | - | - | 68 |
| 17 | - | - | 66 |
| 18 | - | - | 68 |
| 19 | - | - | 70 |
| 20 | - | - | 70 |
