# Supplementary material for: A new ophthalmosaurid ichthyosaur from the Upper Jurassic (Early Tithonian) Kimmeridge Clay of Dorset, UK, with implications for Late Jurassic ichthyosaur diversity
Source: PLoS One. 2020 Dec 9;15(12):e0241700. doi: 10.1371/journal.pone.0241700 (PMC7725355; doi:10.1371/journal.pone.0241700)
Supplement: S3 Table — (DOCX) [file pone.0241700.s004.docx]

S3 Table. Selected pectoral girdle measurements (in mm).

| **Coracoid** |  |
| --- | --- |
| Maximum mediolateral width | 78.7 |
| Maximum anteroposterior length | 97 |
| Length of intercoracoid suture | 60 |
| Length of scapular facet | 40 |
| Length of glenoid facet | 53 |
| **Scapular** |  |
| Maximum proximodistal length | 133 |
| Maximum anteroposterior width – proximal blade | 92 |
| Maximum anteroposterior width – middle of blade | 33 |
| Maximum anteroposterior width – distal blade | 55 |
| Length of coracoid facet | 44 |
| Length of glenoid facet | 43 |
| **Interclavicle** |  |
| Maximum mediolateral width | 129 |
| Maximum length of medial ramus | 95 |
| **Clavicle** |  |
| Maximum anteroposterior width – proximal blade | 33 |
| Maximum anteroposterior width – middle of blade | 31 |
| Maximum anteroposterior width – distal blade­­­ | 5 |
