# Supplementary material for: A new ophthalmosaurid ichthyosaur from the Upper Jurassic (Early Tithonian) Kimmeridge Clay of Dorset, UK, with implications for Late Jurassic ichthyosaur diversity
Source: PLoS One. 2020 Dec 9;15(12):e0241700. doi: 10.1371/journal.pone.0241700 (PMC7725355; doi:10.1371/journal.pone.0241700)
Supplement: S4 Table — (DOCX) [file pone.0241700.s005.docx]

S4 Table. Selected forelimb measurements (in mm).

| **Humerus** |  |
| --- | --- |
| Maximum proximodistal length | 87 |
| Maximum anteroposterior width – proximal end | 65 |
| Maximum anteroposterior width – distal end | 60 |
| Minim anteroposterior width – mid shaft | 45 |
| Length of radial facet | 30 |
| Length of ulna facet | 28 |
| **Radius** |  |
| Maximum proximodistal length | 25 |
| Maximum anteroposterior width | 31 |
| Dorsoventral thickness – proximal | 24 |
| **Ulna** |  |
| Maximum proximodistal length | 26 |
| Maximum anteroposterior width | 34 |
| Dorsoventral thickness – proximal | 32 |
