# Supplementary material for: A new ophthalmosaurid ichthyosaur from the Upper Jurassic (Early Tithonian) Kimmeridge Clay of Dorset, UK, with implications for Late Jurassic ichthyosaur diversity
Source: PLoS One. 2020 Dec 9;15(12):e0241700. doi: 10.1371/journal.pone.0241700 (PMC7725355; doi:10.1371/journal.pone.0241700)
Supplement: S5 Table — (DOCX) [file pone.0241700.s006.docx]

S5 Table. Selected pelvic girdle measurements (in mm).

| **Ischiopubis** |  |
| --- | --- |
| Maximum proximodistal length | 91 |
| Maximum anteroposterior width, proximal end | 32 |
| Maximum anteroposterior width, distal end | 41 |
| Length of the ischiopubic notch | 23 |
